# Abdominal obesity prevalence in Latin America: a systematic review and meta-analysis comparing ATP III and IDF criteria

**DOI:** 10.3389/fendo.2025.1562060

**Published:** 2025-06-17

**Authors:** Víctor Juan Vera-Ponce, Joan A. Loayza-Castro, Fiorella E. Zuzunaga-Montoya, Luisa Erika Milagros Vásquez-Romero, Nataly Mayely Sanchez-Tamay, Juan Carlos Bustamante-Rodríguez, Jhosmer Ballena-Caicedo, Carmen Inés Gutierrez De Carrillo

**Affiliations:** ^1^ Instituto de Investigación de Enfermedades Tropicales, Universidad Nacional Toribio Rodríguez de Mendoza de Amazonas (UNTRM), Amazonas, Peru; ^2^ Facultad de Medicina (FAMED), Universidad Nacional Toribio Rodríguez de Mendoza de Amazonas (UNTRM), Amazonas, Peru; ^3^ Universidad Continental, Lima, Peru

**Keywords:** abdominal obesity, waist circumference, Latin America, prevalence, systematic review, meta-analysis

## Abstract

**Background:**

Abdominal obesity (AO) represents a significant cardiovascular risk factor with distinctive characteristics in Latin American populations. Its prevalence has increased substantially in recent decades, although estimates vary according to the diagnostic criteria.

**Objective:**

To determine the prevalence of AO in Latin American populations through a systematic review with meta-analysis, comparing ATP III and IDF criteria.

**Methods:**

A systematic search was conducted across SCOPUS, Web of Science, PubMed, and EMBASE databases. Observational studies evaluating AO prevalence in Latin American populations using either ATP III (≥102/88 cm) or IDF (≥90/80 cm) criteria were included. Meta-regressions were performed to assess the influence of publication year and sample size.

**Results:**

Sixty-one studies were included (n=281,694 participants). The pooled prevalence according to ATP III criteria was 40% (95% CI: 34-46%) and 62% (95% CI: 56-68%) according to IDF criteria. Sex-stratified analysis revealed significantly higher prevalences in women (ATP III: 50% vs 27%; IDF: 74.3% vs 46.8%). Temporal meta-regression demonstrated an upward trend, particularly in studies utilizing IDF criteria, while sample size showed no significant influence on prevalence estimates. Substantial geographic variations were observed, with Mexico and Venezuela exhibiting the highest prevalences. Heterogeneity was considerably high (I²>99%) across all analyses.

**Conclusions:**

AO prevalence in Latin America is high and demonstrates significant sexual dimorphism. These findings challenge the validity of current cut-off points and suggest the need to develop Latin American-specific criteria based on clinically relevant outcomes.

## Introduction

Abdominal obesity (AO), characterized by excessive visceral fat accumulation in the abdominal region, represents one of the major global public health challenges. This specific pattern of body fat distribution has been recognized as an independent risk factor for cardiovascular diseases, type 2 diabetes, and metabolic syndrome. According to the World Health Organization, obesity prevalence has tripled since 1975, being particularly concerning in developing regions such as Latin America (1).

In the Latin American context, the nutritional transition experienced over recent decades has significantly contributed to the increase in AO. Changes in traditional dietary patterns towards hypercaloric diets rich in saturated fats and refined sugars and decreased physical activity have created an obesogenic environment affecting all socioeconomic strata (2).

The measurement of AO, primarily through waist circumference (WC), has become a fundamental tool for cardiometabolic risk assessment in clinical practice. Specific cut-off points for Latin American populations have been subject to debate, considering the region’s particular ethnic and anthropometric differences. Previous studies have suggested that reference values may differ from those established for European or North American populations (3).

The economic and social implications of AO in Latin America are substantial. Direct and indirect costs associated with treating its complications represent a significant burden for the region’s healthcare systems, which frequently operate with limited resources. It is estimated that costs related to obesity and its comorbidities may represent between 0.7% and 2.8% of the gross domestic product of Latin American countries (4).

Despite the problem’s relevance, there is significant heterogeneity in data regarding AO prevalence in Latin America, which hinders a precise understanding of the problem’s magnitude and the development of effective interventions. The variability in measurement methodologies, diagnostic criteria used, and studied population characteristics necessitates a systematic review (SR) to consolidate available evidence and provide more accurate regional prevalence estimates (5).

## Methods

### Study design

A SR with a meta-analysis of observational studies evaluating AO prevalence in Latin American populations was conducted. The research protocol was developed following the PRISMA (Preferred Reporting Items for Systematic Reviews and Meta-Analyses) guidelines (6), adapted according to specific methodological recommendations for systematic reviews of prevalence studies proposed by Munn et al. (7).

### Search strategy

The systematic literature search was conducted across four major electronic databases: SCOPUS, Web of Science (WOS), including the SciELO Citation Index catalog, PubMed/MEDLINE, and EMBASE. The selection of these databases followed the recommendations of the Cochrane Handbook for Systematic Reviews of Interventions, which suggests utilizing multiple databases to maximize search comprehensiveness (8). The search strategy was constructed by combining three groups of terms using Boolean operators: (1) terms related to AO measurement (“waist circumference,” “abdominal obesity,” “central obesity”), and (3) Latin American country names and regional terms (“Latin America,” “South America,” “Central America,” “Argentina,” “Brazil,” “Chile,” etc.). The detailed search strategy for each database, including all terms, Boolean operators, and filters used, is available in **Supplementary Data Sheet 1**.

### Selection criteria

Predefined eligibility criteria were established for study selection. To be included, studies had to meet the following criteria: (1) observational design, primarily cross-sectional; (2) report AO prevalence data; (3) use standardized diagnostic criteria according to either Adult Treatment Panel III (ATP III) (9) or International Diabetes Federation (IDF) (10) definitions; (4) evaluate general Latin American populations; (5) employ probabilistic sampling; and (6) be published in any language. Studies were considered eligible regardless of their publication date.

Studies were excluded if they: (1) evaluated specific populations (e.g., patients with specific comorbidities, particular occupational groups, or selected clinical populations); (2) were case reports; (3) were letters to the editor; (4) were systematic or narrative reviews; (5) were bibliometric studies; (6) used diagnostic criteria different from ATP III or IDF; and (7) used non-probabilistic sampling or lacked a clear description of sampling methodology.

### Study selection process

The search strategy was implemented across selected databases, and results were imported into Rayyan QCRI software, a web-based platform specifically designed for conducting SR. Two independent reviewers (VJVP and JALC) performed the selection process simultaneously and were blinded, following a previously established protocol. The selection process was conducted in two phases: screening titles and abstracts and subsequently through full-text review of potentially eligible articles.

After completing the independent review, the blind mode in Rayyan was lifted to identify concordances and discrepancies between reviewers. Selection discrepancies were discussed between the two primary reviewers seeking consensus. In cases where consensus could not be reached, a third reviewer (FEZM) intervened to decide on the inclusion or exclusion of the study in question.

### Data extraction

A standardized template for systematic data extraction was developed using Microsoft Excel 2023. Two reviewers (LEMVR and NMST) performed the extraction independently, following the same consensus protocol used in the selection phase. In cases of disagreement, a third reviewer (JCBR) intervened to resolve discrepancies.

Extracted data encompassed detailed information on the bibliometric characteristics of studies, including author(s), publication year, and Latin American country(ies) where the research was conducted. Fundamental methodological characteristics, such as study design, data collection period, and sampling method employed, were also compiled. To ensure a comprehensive evaluation of the studied population, data were extracted on sample size, sex distribution, age range with measures of central tendency, and other relevant demographic characteristics.

Particular emphasis was placed on extracting technical aspects of AO measurement and diagnosis. This included detailed documentation of the diagnostic criteria employed (ATP III or IDF) and the specific methodology used for WC measurement, considering these variables are fundamental for subsequent synthesis and analysis of results.

### Risk of bias assessment

Two reviewers (JBC and GIGDC) independently assessed the risk of bias for included studies, utilizing the methodological tool proposed by Munn et al. (7) for prevalence studies. This tool was selected for its robustness and specificity in evaluating prevalence studies within the context of systematic reviews and its capacity to examine critical methodological aspects in epidemiological studies.

The assessment was structured around ten fundamental criteria: (1) sample representativeness for the studied Latin American population, (2) appropriateness of the sampling frame used, (3) randomization in participant selection, (4) adequate handling of non-response rate, (5) direct WC measurement by trained personnel, (6) standardized application of AO diagnostic criteria (ATP III or IDF), (7) use of validated and calibrated measuring instruments, (8) consistency in anthropometric measurement protocol, (9) appropriate data collection period, and (10) adequate prevalence calculation. Each criterion was evaluated as “Low risk,” “High risk,” or “Unclear,” following the tool’s specific guidelines.

The final risk of bias rating was determined through a scoring system where each criterion rated as “Low risk” received one point. The overall risk classification was established in three categories: high risk (0–3 points), moderate risk (4–6 points), and low risk (7–10 points). In cases of discrepancy between evaluators, differences were resolved through discussion and consensus, with the intervention of a third reviewer when necessary.

### Statistical analysis

All quantitative analyses were performed using R software (version 4.2.2). Studies were included if they reported AO prevalence based on ATP III or IDF definitions, specifying both the total number of participants (n) and the identified cases (r). We utilized the ‘meta’ package—specifically its ‘metaprop’ function—to conduct the analyses. Proportions were transformed using the Freeman–Tukey double arcsine method (sm = “PFT”), which is advantageous for stabilizing variance when values are near the distribution’s extremes.

Exact confidence intervals for these proportions were generated through the Clopper–Pearson method (method.ci = “CP”). In light of the substantial heterogeneity anticipated across various Latin American populations—stemming from differences in waist-circumference measurement techniques and diagnostic standards—a random-effects approach was selected, following DerSimonian and Laird.

Given that AO diagnostic cutoffs (as per ATP III and IDF) exhibit sex-related variations in Latin American cohorts, we performed sex-stratified meta-analyses. This approach enabled the computation of distinct prevalence estimates for males and females, using the aforementioned statistical procedures. The Q test for heterogeneity between subgroups helped determine whether any observed prevalence gaps by sex were statistically meaningful.

We assessed overall heterogeneity with the I² statistic and Cochran’s Q test. The Hartung–Knapp adjustment was applied to generate more conservative and reliable confidence intervals. Meta-analysis outcomes were then reported alongside their corresponding 95% confidence intervals and visualized using forest plots. Additional pre-specified subgroup analyses considered sex, diagnostic criteria (ATP III vs. IDF), and geographic regions within Latin America.

Furthermore, meta-regressions were conducted using the ‘metafor’ package to examine possible sources of heterogeneity. The variables investigated included publication year, sample size, and the methodological quality of the studies. Each meta-regression was run under a mixed-effects model using weighted least squares, in which weights were inversely related to each study’s variance. Results from these meta-regressions were illustrated in bubble plots, where the size of each bubble indicated the relative weight of that study in the analysis.

## Results

**Figure 1:**
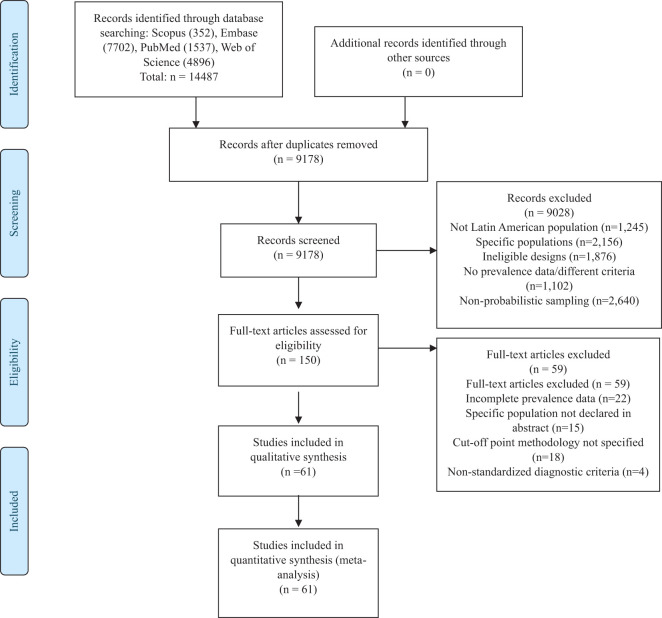
Flowchart of study selection.

A total of 9,148 records were initially identified; 9,028 were excluded after screening titles and abstracts, mainly because they did not include Latin American populations or focused on specific populations. Out of 120 full-text articles evaluated, 59 were removed for reasons such as incomplete prevalence data and unspecified methodology. Ultimately, 61 studies fulfilled the eligibility criteria and were incorporated into the systematic review and meta-analysis (11–71). A detailed overview of this process is provided in **Figure 1**.

### Main characteristics

The systematic search identified 61 studies evaluating AO prevalence in Latin American populations, as shown in **Supplementary Data Sheet 2**, spanning a publication period from 2005 to 2024. Collectively, these studies included a cumulative sample of 297,794 participants distributed across 10 countries in the region: Brazil (n=30 studies) (12, 13, 16, 17, 21, 23–25, 27, 30, 31, 33, 36, 39, 41, 44, 45, 48–50, 52, 55, 58, 60, 65–68, 70, 71), Mexico (n=8) (20, 32, 34, 42, 59, 61, 64, 69), Peru (n=9) (14, 15, 18, 29, 47, 51, 54, 62, 63), Colombia (n=6) (19, 22, 38, 43, 56, 57), Venezuela (n=2) (11, 26), Chile (n=2) (35, 53), Puerto Rico (n=2) (28, 37), Ecuador (n=1) (46), and Guatemala (n=1) (40). Brazil emerged with the highest scientific output, representing 44.3% of included studies.

Most studies employed a cross-sectional design, with only five cohort or longitudinal studies (35, 45, 47, 51, 61). Sample size varied considerably among studies, from local investigations with 102 participants (Mohanna) (14) to national studies including 68,288 participants (Higuita) (56). The proportion of women in the samples ranged from 28.97% to 100%, with a median of 59.3%. Nineteen studies reported participants’ mean age ranging from 21.4 to 77.7 years.

Regarding measurement methodology, considerable homogeneity was observed in the WC measurement protocol. Most studies (85.2%) specified using a non-extensible measuring tape and the anatomical measurement point, the most common being the midpoint between the last rib and the iliac crest. However, some studies used alternative anatomical references, such as the umbilical level or the narrowest point of the torso.

Diagnostic criteria for AO were distributed between those established by ATP III (11–18, 23–25, 27–29, 32, 36, 37, 39, 41, 42, 45, 49, 52, 53, 61, 66, 70) and IDF criteria specific for Latin American populations (19, 21, 22, 30, 31, 33, 34, 38, 40, 43, 44, 46–48, 50, 51, 55–60, 62–65, 68, 69, 71). Some studies (14.8%) evaluated prevalence using both criteria, allowing direct comparison between different definitions (20, 26, 35, 54).

### Bias analysis

The risk of bias assessment for the 61 included studies, shown in **Supplementary Data Sheet 3**, conducted using Munn et al.’s tool (7), revealed that most studies (55 studies, 90.2%) demonstrated a low risk of bias (score ≥7). In comparison, only 6 studies (9.8%) were classified as moderate risk (score = 6). The most frequently fulfilled criteria were an appropriate sampling frame and adequate statistical analysis in almost all studies. Measurement methods were considered valid in studies that followed any of the three standardized protocols for WC measurement: World Health Organization/STEPwise (WHO/STEPS), National Institutes of Health (NIH), or Multi-Ethnic Study of Atherosclerosis (MESA).

Conversely, the main limitations identified were inadequate sample size in some studies and, to a lesser extent, response rate and handling. Studies classified as moderate risk (Patiño 2011, Muñoz 2014, Mulatinho 2018, Rodrigues 2023, Do Nascimento 2023) (22, 37, 48, 65, 66) primarily showed deficiencies in these aspects. However, it is important to note that no study was classified as high risk of bias.

### Funnel plot analysis

The funnel plots, presented in **Supplementary Data Sheet 4**, show the distribution of studies according to their precision (standard error) and Freeman-Tukey transformed proportion. For ATP III (A), studies are distributed relatively symmetrically around the central line, although some studies deviate from the expected pattern, particularly at higher proportions. Most studies concentrate on the proportion range between 0.4 and 0.8, with standard errors varying primarily between 0.01 and 0.03.

For IDF (B), the distribution shows a more dispersed and asymmetric pattern, with studies extending across a wider range of transformed proportions (0.6 to 1.2). A higher concentration of studies is observed in higher proportions, consistent with IDF’s more inclusive criteria. In both plots, asymmetry, and point dispersion suggest possible heterogeneity and potential publication bias.

### Meta-analysis of AO prevalence - ATP III

**Figure 2:**
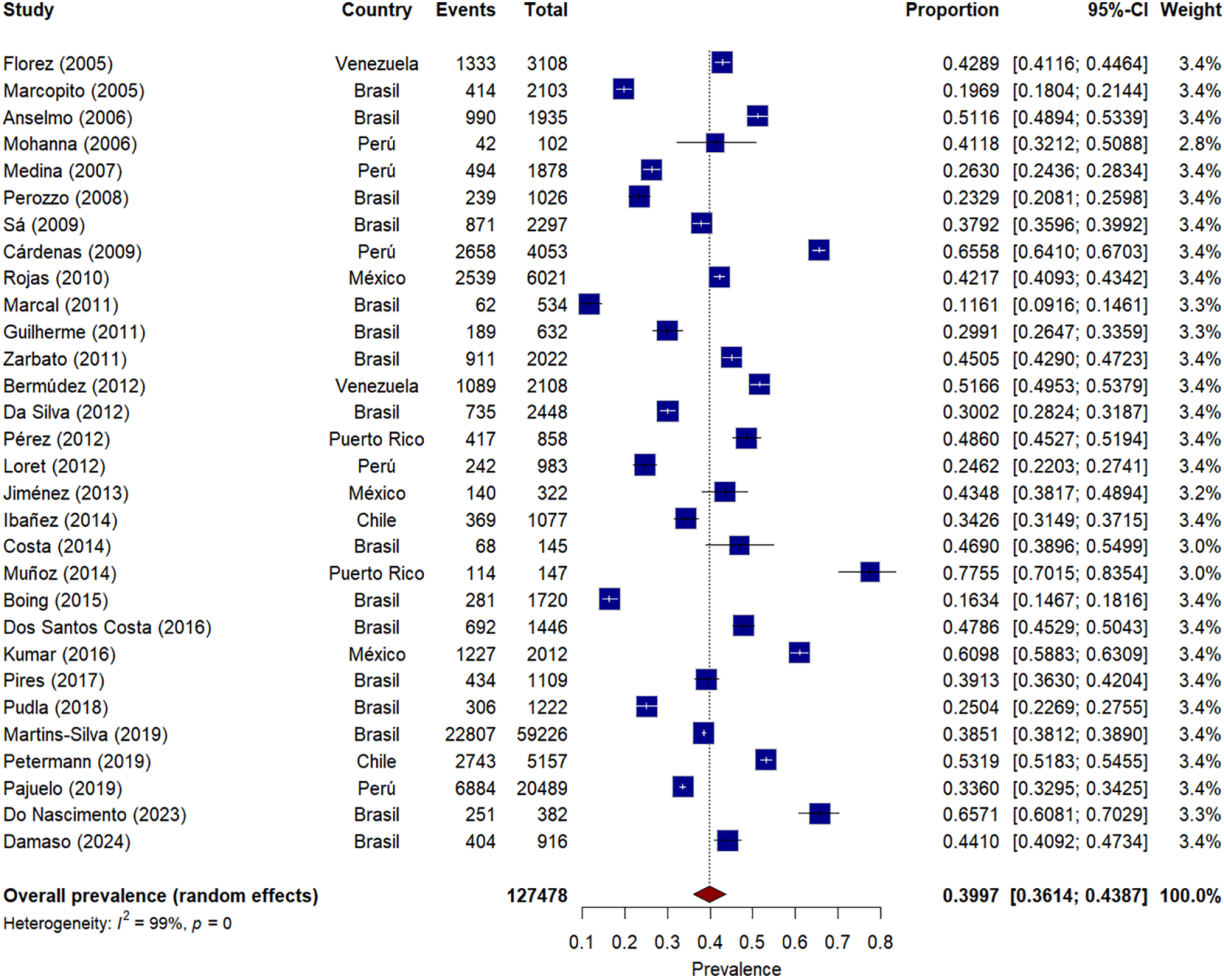
Forest plot* of AO prevalence meta-analysis according to ATP III criteria in Latin America. * The squares represent the point prevalence of each study, while the horizontal lines indicate the 95% confidence intervals. The size of the squares is proportional to the relative weight of each study in the meta-analysis. The diamond at the bottom represents the combined prevalence estimate with its 95% confidence interval.

The meta-analysis of AO prevalence according to ATP III criteria included 30 studies, **Figure 2**, with a total sample of 127,478 participants from eight Latin American countries. The prevalence pooled 40% (95% CI: 34 - 46%). Significant heterogeneity was observed among studies (I² = 99%, p < 0.001).

Individual prevalences varied widely across studies, ranging from 12% (Marcal, 2011) (23) to 78% (Muñoz, 2014) (37). The studies carrying the greatest weight in the meta-analysis were conducted in Brazil (Martins-Silva, 2019; n=59,226) (52) and Peru (Pajuelo, 2019; n=20,489) (54), contributing significantly to the overall estimate due to their large sample sizes.

By country, interesting patterns were observed: Venezuela showed consistently high prevalences in its two studies (Florez, 2005: 43%; Bermúdez, 2012: 52%) (11, 26), while Brazil, the country with the highest number of included studies, presented notable variability in its estimates (from 16% in Boing, 2015 to 48% in Dos Santos Costa, 2016) (39, 41). Chile reported moderately high prevalences in its two studies (Ibañez, 2014: 34%; Petermann, 2019: 53%) (35, 53).

### Meta-analysis of AO prevalence - IDF

**Figure 3:**
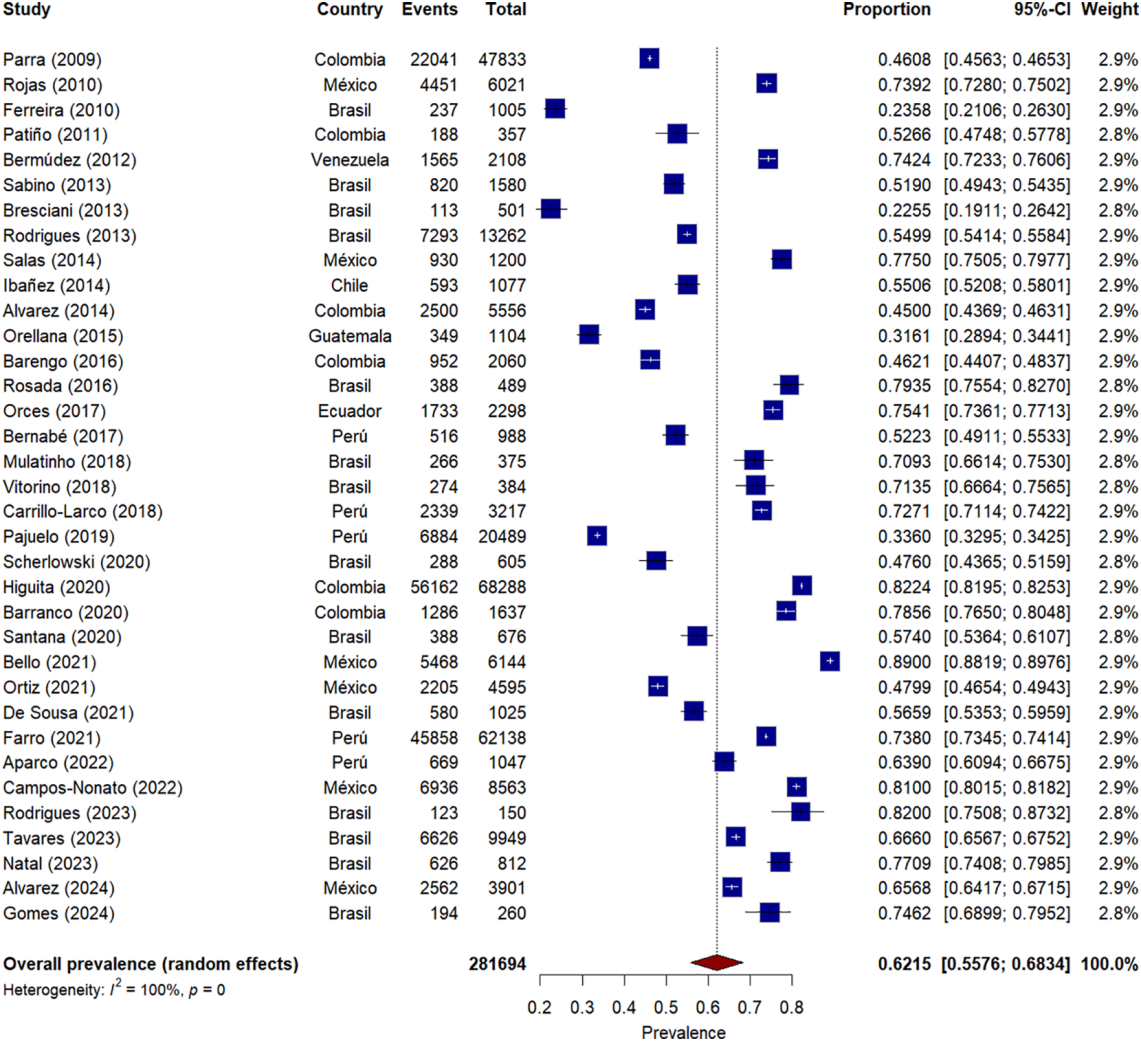
Forest plot* of AO prevalence meta-analysis according to IDF criteria in Latin America, * The squares represent the point prevalence of each study, while the horizontal lines indicate the 95% confidence intervals. The size of the squares is proportional to the relative weight of each study in the meta-analysis. The diamond at the bottom represents the combined prevalence estimate with its 95% confidence interval.

The meta-analysis of AO prevalence according to IDF criteria included 34 studies, as shown in **Figure 3**, with a total sample of 281,694 participants from nine Latin American countries. The pooled prevalence was significantly higher than that found with ATP III criteria, reaching 62% (95% CI: 56 - 68%). Heterogeneity between studies was very high (I² = 100%, τ² = 0.0382, p < 0.001), suggesting important variability in estimates.

Individual prevalences ranged from 23% (Bresciani, 2013) (31) to 89% (Bello, 2021) (61). The largest studies, which contributed greater weight to the meta-analysis, were conducted in Colombia (Higuita, 2020; n=68,288) (56) and Peru (Farro, 2021; n=62,138) (62), reporting prevalences of 82% and 74%, respectively.

Geographic analysis revealed distinctive patterns. Mexico consistently showed high prevalences across its six studies (48%-89%) (20, 32, 34, 42, 59, 61, 64, 69), with an increasing trend in more recent studies. Colombia presented considerable variability between studies (45%-82%) (19, 22, 38, 43, 56, 57), although more recent and larger sample size studies reported prevalences above 75%. Brazil, with the highest number of studies (n=13) (12, 13, 16, 17, 21, 23–25, 27, 30, 31, 33, 36, 39, 41, 44, 45, 48–50, 52, 55, 58, 60, 65–68, 70, 71), showed wide variation in its estimates (23%-82%), possibly reflecting regional and temporal differences.

### Meta-analysis of AO prevalence by sex - ATP III

A sex-stratified analysis revealed substantial differences in AO prevalence according to ATP III criteria. In men (n=36,376), the pooled prevalence was 27.16% (95% CI: 20.10 - 34.84%), while in women (n=49,874) it was significantly higher, reaching 49.91% (95% CI: 53.64 - 56.18%). Both analyses showed significant heterogeneity (I² = 99%).

In the male population, individual prevalences showed wide variation, ranging from 1% (Marcal, 2011) (23) to 50% (Petermann, 2019) (53). Studies with larger sample sizes, such as Martins-Silva (2019), with 25,920 participants, reported prevalences around 21%, suggesting that smaller sample sizes might influence more extreme estimates. In contrast, among women, the range of prevalences was narrower but equally heterogeneous, varying from 23% (Perozzo, 2008) (16) to 70% (Do Nascimento, 2023) (66). The largest study in women (Martins-Silva, 2019; n=33,306) (52) reported a prevalence of 52%, very close to the pooled estimate.

Furthermore, the prevalence difference between sexes remained consistent across countries. For example, in Chile (Ibañez, 2014; Petermann, 2019) (35, 53), the prevalence in women (64% and 55%) was approximately double that in men (31% and 50%). This trend was similarly observed in other countries, although with variability.

### Meta-analysis of AO prevalence by Latin American country

**Figure 4:**
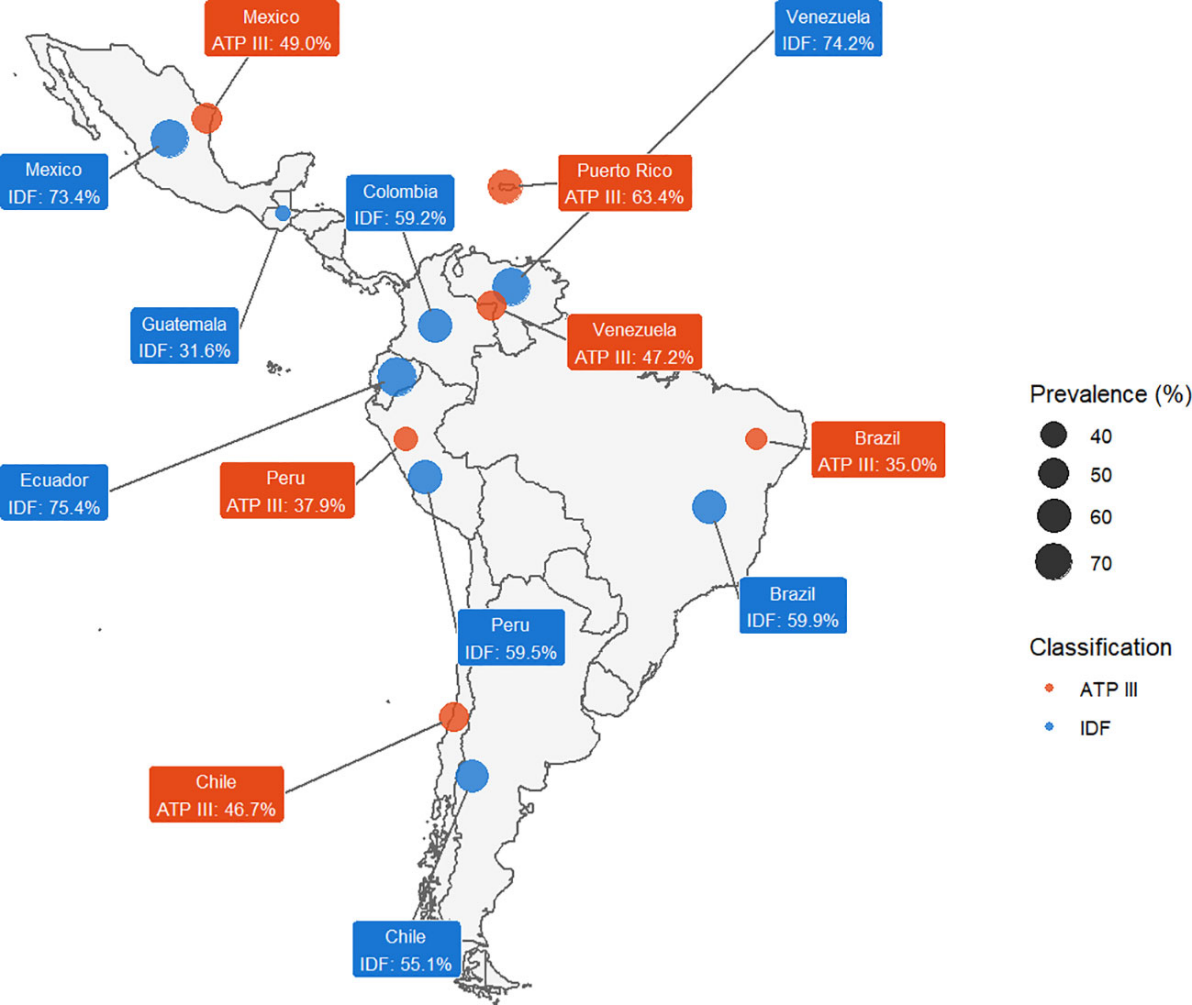
Map of Latin America with the prevalence of LA according to ATP III and IDF.

The sensitivity analysis stratified by country, presented in **Table 1** and **Figure 4**, revealed distinctive geographic patterns according to study location. Under ATP III criteria, Puerto Rico showed the highest prevalence (63.44%, 95% CI: 34.02-88.23%), followed by Mexico (48.96%, 95% CI: 35.03-62.97%) and Venezuela (47.24%, 95% CI: 38.72-55.85%). In contrast, Brazil, with the highest number of studies (n=16), presented the lowest prevalence (34.96%, 95% CI: 29.91-40.19%). All country-specific analyses showed very high heterogeneity (I² > 97%).

**Table 1:** Sensitivity analysis of AO prevalence by ATP III and IDF criteria according to Latin American country.

Classification	Country	Number of studies	Prevalence	95% CI	I²
ATPIII	Venezuela	2	47.24	38.72 – 55.85	97.4%
	Brazil	16	34.96	29.91 – 40.19	99.1%
	Peru	5	37.89	22.65 – 54.45	99.8%
	Mexico	3	48.96	35.03 – 62.97	99.1%
	Puerto Rico	2	63.44	34.02 – 88.23	97.8%
	Chile	2	46.66	25.88 – 62.33	99.2%
IDF	Colombia	6	59.19	38.58 – 78.24	100%
	Mexico	6	73.41	61.04 – 84.15	99.8%
	Brazil	14	59.92	52.90 – 66.74	99.2%
	Venezuela	1	74.24	72.35 – 76.09	–
	Chile	1	55.06	52.08 – 58.02	–
	Guatemala	1	31.61	28.90 – 34.39	–
	Ecuador	1	75.41	73.63 – 77.15	–
	Peru	5	59.54	36.69 – 80.39	100%

Under IDF criteria, prevalences were consistently higher across all countries. Mexico led with a prevalence of 73.41% (95% CI: 61.04-84.15%), closely followed by Ecuador (75.41%, 95% CI: 73.63-77.15%) and Venezuela (74.24%, 95% CI: 72.35-76.09%), although the latter two had only one study each. Guatemala presented the lowest prevalence (31.61%, 95% CI: 28.90-34.39%). Brazil, Colombia, and Peru showed similar prevalences, around 60%. Heterogeneity remained very high (I² > 99%) in all countries with multiple studies.

### Meta-analysis of AO prevalence by sex - IDF

Sex-stratified analysis according to IDF criteria in **Table 2** also revealed marked differences between men and women. In the male population (n=56,800), the pooled prevalence was 46.8% (95% CI: 38.9% - 54.8%), while in women (n=93,124) the prevalence was significantly higher, reaching 74.3% (95% CI: 65.3% - 82.3%). Both analyses showed high heterogeneity (I² = 100%), reflecting considerable between-study variability.

**Table 2:** Sensitivity analysis of MetS prevalence by ATP III and IDF criteria stratified by sex in Latin America.

Classification	Sex	Number of studies	Prevalence	95% CI	I²
ATPIII	Male	14	27.16	20.10 – 34.84	99%
	Female	17	49.91	43.64 – 56.18	99%
IDF	Male	17	43.20	34.88 – 51.71	100%
	Female	19	74.27	65.26 – 82.34	100%

Individual prevalences in men ranged from 12.7% (Orellana, 2015, Guatemala) (40) to 73.9% (Campos-Nonato, 2023, Mexico) (64). Studies carrying greater statistical weight due to their sample size, such as Parra (2009) (19) in Colombia (n=17,937) and Farro (2021) in Peru (n=26,781), reported prevalences of 38.8% and 61.1%, respectively, suggesting important geographic variations.

The prevalence range in women was higher and less dispersed, varying from 50.2% (Parra, 2009, Colombia) (19) to 88.0% (Barranco, 2020, Colombia) (57). The largest studies, such as Farro (2021) (62) in Peru (n=35,357) and Parra (2009) (19) in Colombia (n=29,896), showed prevalences of 85.1% and 50.2%, respectively.

Notably, the prevalence difference between sexes remained consistent across all countries, although with varying magnitudes. For example, in Mexico, both Salas (2014) (34) and Campos-Nonato (2022) (64) reported differences of approximately 20 percentage points between men and women. This sex disparity was even more pronounced than that observed with ATP III criteria despite IDF cut-off points being lower for both sexes.

### Meta-regression of AO prevalence - ATP III by country and sample size

**Figure 5:**
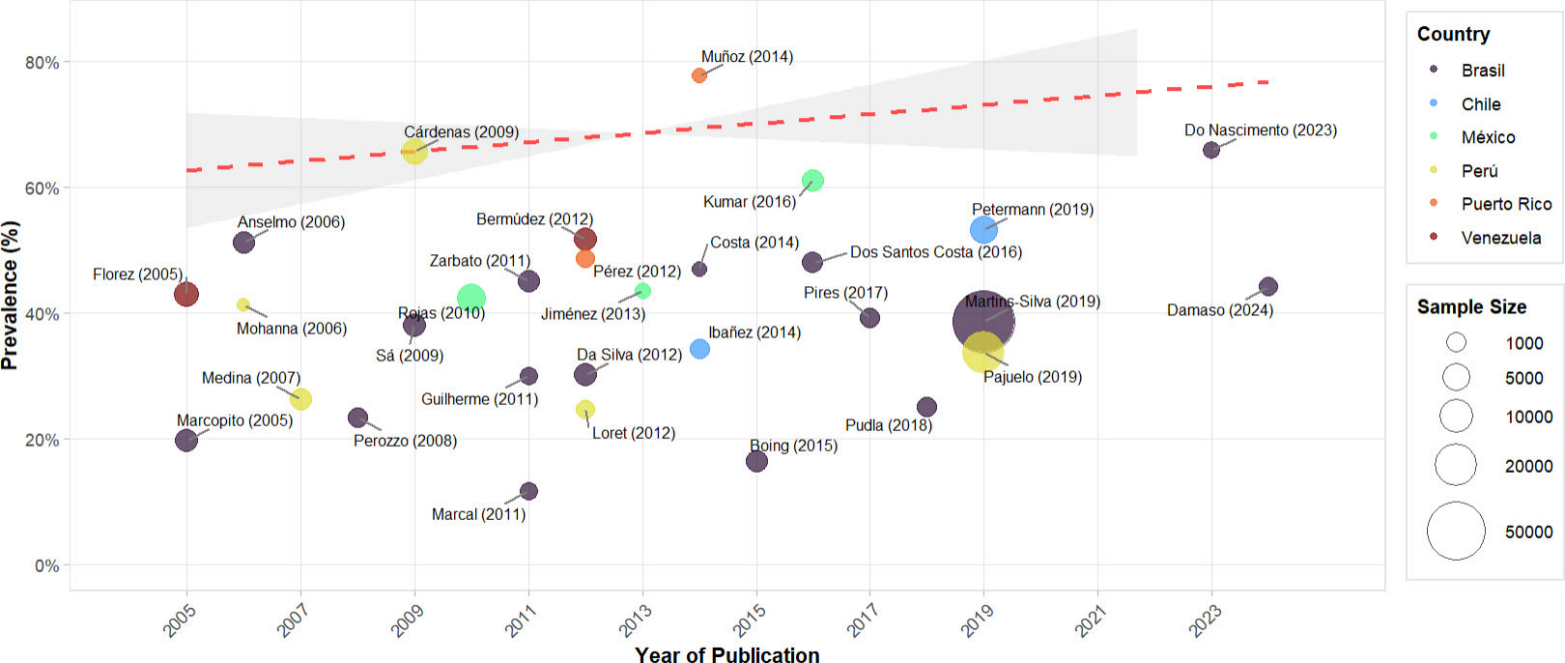
Meta-regression of the prevalence of AO – ATP III by year of publication in Latin American countries.

Meta-regression analysis (**Figure 5**) revealed an upward temporal trend in AO prevalence in Latin America during 2005-2024, according to ATP III criteria. The trend line shows a modest but sustained increase, from approximately 60% at the beginning of the period to nearly 75% in recent years. This trend persists despite considerable heterogeneity observed between studies, as evidenced by the dispersion of points around the trend line.

The visualization, which incorporates sample size through circle diameter and differentiates countries by colors, shows that larger studies, such as Martins-Silva (2019) (52) and Pajuelo (2019) (54), tend to cluster near the central trend line. Notable outliers were identified, such as Muñoz (2014) (37) with an unusually high prevalence and Marcal (2011) (23) with a markedly low prevalence, although these did not significantly alter the general upward trend. Brazil contributed the largest number of studies, followed by Peru, providing a more complete temporal representation of these regions.

The meta-regression analysis by sample size (**Supplementary Data Sheet 5**) shows a slightly downward trend in AO prevalence according to ATP III criteria, as observed in the red dotted line. The logarithmic scale on the X-axis allows better visualization of the relationship between studies with vastly different sample sizes, ranging from approximately 100 to more than 10,000 participants.

Interestingly, studies with smaller samples tend to report more variable prevalences, as evidenced by the extremes of Muñoz (2014) (37) with nearly 80% and Marcal (2011) (23) with approximately 12%. In contrast, studies with larger sample sizes, such as Martins-Silva (2019) (52) and Pajuelo (2019) (54), tend to converge toward more moderate prevalences, around 35-40%. This observation suggests possible publication bias in small studies or greater precision in estimates from larger studies. However, significant heterogeneity persists even in large samples, as indicated by the shaded confidence area that remains wide across the entire spectrum of sample sizes.

### Meta-regression of AO prevalence - IDF by country and sample size

**Figure 6:**
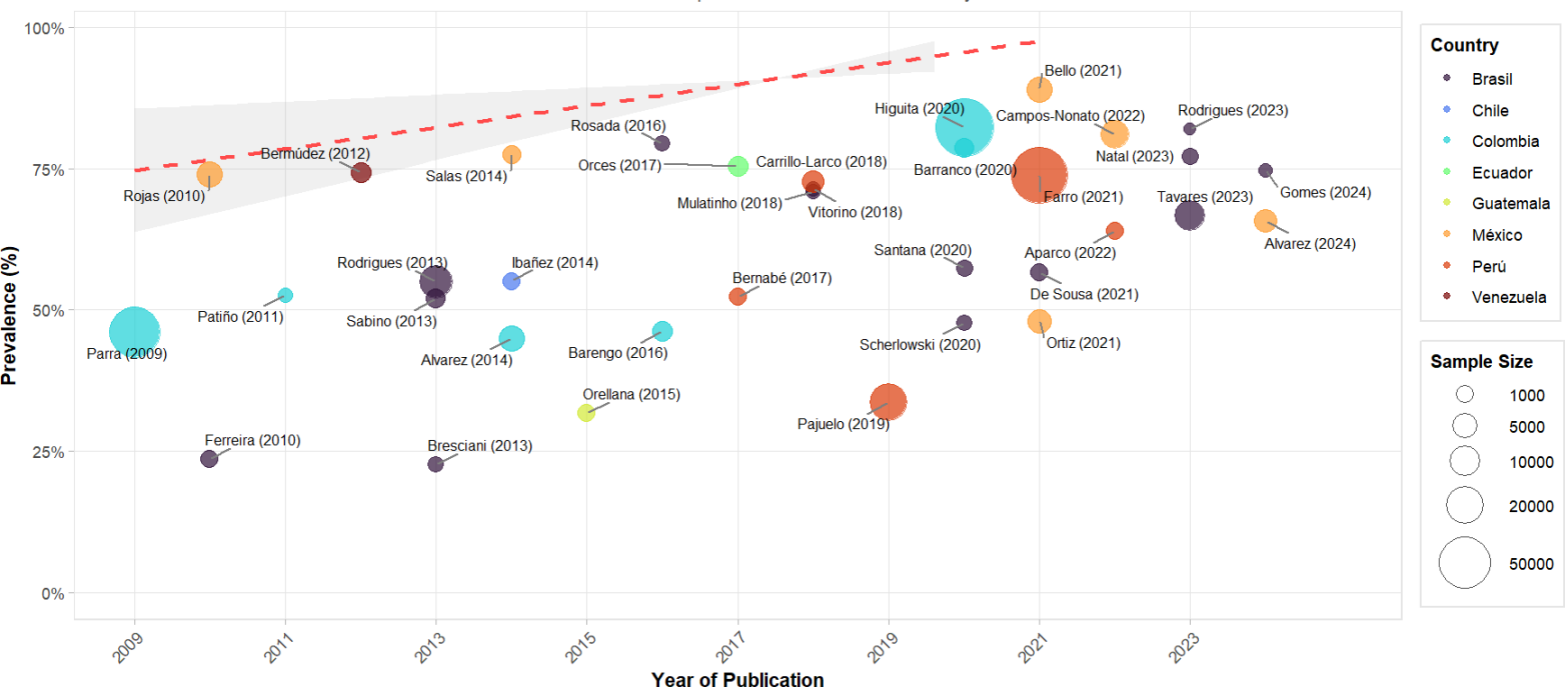
Meta-regression of the prevalence of OA – IDF by year of publication in Latin American countries.

The temporal meta-regression analysis for AO prevalence according to IDF criteria shows a clear upward trend during 2009-2024, as evidenced by the red dotted line (**Figure 6**). Prevalence increased from approximately 75% in 2009 to nearly 95% in more recent studies, with a steeper slope than in the ATP III analysis. Larger studies, represented by larger circles, such as Higuita (2020) (56) in Colombia and Farro (2021) (62) in Peru, tend to report prevalences above 80%.

Notable geographic variability is observed, with Mexico and Colombia consistently showing higher prevalences, while Brazil demonstrates greater dispersion in its estimates. More recent studies (2020-2024) tend to converge toward higher prevalences, regardless of country of origin, suggesting a possible real increase in AO prevalence in the region when using IDF criteria. This upward temporal trend remains robust even considering between-study heterogeneity, as indicated by the shaded confidence area.

Meta-regression by sample size for studies using IDF criteria (**Supplementary Data Sheet 6**) shows a relatively stable trend, as observed in the red dotted line that remains around 60% across different sample sizes. The logarithmic scale on the X-axis allows visualization that studies with smaller samples (less than 1,000 participants) show greater variability in reported prevalences. In comparison, larger studies (more than 10,000 participants) tend to show more consistent estimates, although with some notable exceptions, such as Higuita (2020) (56) and Farro (2021) (62) reporting substantially different prevalences despite their large sample sizes. This distribution suggests that sample size alone does not explain the observed heterogeneity in AO prevalence estimates according to IDF criteria in Latin America.

## Discussion

### Main findings

This systematic review with meta-analysis identified a high prevalence of AO in the Latin American population, with marked differences according to the diagnostic criteria used. Under ATP III criteria, the pooled prevalence was 40% (95% CI: 34-46%), while with IDF criteria, which are more specific to the Latin population and have lower cut-off points, the prevalence increased significantly to 62% (95% CI: 56-68%). This substantial difference in estimates according to diagnostic criteria highlights the importance of considering population-specific cut-off points for Latin Americans when assessing cardiometabolic risk.

Sex-stratified analysis revealed significant dimorphism, with higher prevalence in women than in men under both diagnostic criteria. According to ATP III, prevalence in women was 50% (95% CI: 41-59%) versus 27% (95% CI: 18-38%) in men, while with IDF criteria these differences persisted but with higher prevalences: 74.3% (95% CI: 65.3-82.3%) in women and 46.8% (95% CI: 38.9-54.8%) in men. Temporal analysis showed an increasing trend in prevalence during the 2005–2024 period, particularly notable in studies using IDF criteria, suggesting a real increase in AO in the region. Furthermore, important geographic variations were observed, with Mexico and Venezuela presenting the highest prevalences under both diagnostic criteria.

### Comparison with literature

Our findings on AO prevalence in Latin America are consistent with previous systematic reviews, although showing slightly higher prevalences. Our results reveal a high prevalence of AO in the studied population, with notable differences according to the diagnostic criteria employed. The overall prevalence of this condition is 40% according to ATP III and 62% according to IDF. When comparing our results with those of other world regions, we observe that the prevalence in Latin America is considerably higher than that reported in Asian countries, where a recent meta-analysis found a prevalence of 40.8% according to ATP III criteria (72). In the United States, Sun et al. (73) found that AO prevalence increased from 35.48 to 53.13%, which resembles our findings under ATP III criteria.

The significant differences between sexes deserve special attention, with women showing substantially higher prevalences under both ATP III (50% vs 27%) and IDF criteria (74.3% vs 46.8%). These findings are consistent with those reported by Wagner et al. (74) in Europe, where prevalences varied between 20.7-36.2% in men and 24.6-46.8% in women. Similarly, Gutiérrez‐Fisac et al. (75) found a marked difference between sexes in Spain, with prevalences of 35.5% in men and 61.4% in women. This disparity notably contrasts with observations in Asia, where Xi et al. (76) reported a significant increase in AO prevalence in China, from 8.5% in 1993 to 27.8% in 2009, although with smaller differences between sexes. Additionally, this marked sex difference observed in our study coincides with findings from the CARMELA study (Cardiovascular Risk Factor Multiple Evaluation in Latin America), which evaluated seven major Latin American cities and found significantly higher prevalences in women than in men (77).

### Methodological considerations

The methodological evaluation of the included studies revealed considerable variability in WC measurement methods. Of all analyzed studies, only 36 followed standardized protocols: 30 used the WHO/STEPS protocol, three used the NIH protocol, and three used MESA. This methodological variability could significantly influence prevalence estimates, as previous studies have shown that different measurement points can result in variations of up to 5 cm in WC (78).

Different diagnostic criteria (ATP III and IDF) substantially impacted prevalence estimates. IDF criteria, with lower cut-off points specific to the Latin American population (≥90 cm for men and ≥80 cm for women), resulted in significantly higher prevalences than ATP III criteria (≥102 cm for men and ≥88 cm for women). This difference highlights the importance of considering population-specific criteria, as noted by various international consensuses (79).

These findings raise serious questions about the validity of current cut-off points for the Latin American population. The prevalences found (40% with ATP III and 62% with IDF) are notably higher than the traditionally expected obesity prevalence of around 30%, suggesting possible risk overestimation (1). This discrepancy is particularly concerning considering that current cut-off points were primarily derived from correlation with body mass index (BMI), where 90/80 cm correlated with overweight (BMI ≥25) and 102/88 cm with obesity (BMI ≥30) (80, 81), without adequately considering the specific anthropometric characteristics of the Latin American population or their direct relationship with cardiometabolic risk (3). This simplistic approach of translating BMI-based cut-off points to WC might not be the most appropriate, suggesting the urgent need to develop Latin American-specific cut-off points based on clinically relevant outcomes rather than simple anthropometric correlations.

Furthermore, it is important to note considerations regarding AO measurement methods; in 2008, the World Health Organization (WHO) published a report with recommendations for measuring WC and Waist-Hip ratio, emphasizing the anatomical placement of the measuring tape and factors that could modify such measurements. Two standardized methods are primarily mentioned (82). The WHO STEPs protocol describes the first, establishes that WC measurement should be taken at the midpoint between the lower margin of the last palpable rib and the top of the iliac crest. In contrast, the United States National Institutes of Health (NIH) protocol for measuring obesity recommends measuring at the level of the superior border of the iliac crest. Additionally, the NIH established a third protocol, the “Multi-Ethnic Study of Atherosclerosis,” which suggests taking measurements at the umbilical region; however, this method was considered an underestimation of the true WC measurement (83).

Indeed, the analysis of WC measurement methodology across the 61 included studies revealed that some followed internationally recognized standardized protocols. The majority of these (30 studies) adopted the WHO/STEPS protocol (13, 14, 16–19, 21, 24, 25, 27, 29–32, 34, 35, 38, 39, 41, 46, 49, 55, 58–60, 68–71), which measures at the midpoint between the last rib and iliac crest. Three studies used the NIH protocol (measurement at the superior border of the iliac crest) (26, 28, 40), and another three employed the umbilical method according to the MESA protocol (11, 15, 36).

The remaining 25 studies did not specify their measurement methodology or provide sufficient details to determine adherence to any standardized protocol. An interesting temporal trend was observed: older studies (2005-2015) tended to adhere more consistently to standardized protocols, while several recent studies, especially those based on national health surveys, did not specify their measurement methodology.

These factors would be responsible for methodological variability, which, while representing an important limitation in interpreting the meta-analysis results, also reveals how the lack of measurement standardization could contribute to the observed heterogeneity in prevalence estimates across studies, especially considering that the measurement point can significantly affect the classification of individuals as AO cases.

### Public health implications

The findings of this systematic review have important implications for public health in Latin America. Regardless of the criteria used, the high prevalence of AO suggests a public health crisis requiring urgent attention. This situation is particularly alarming, considering that AO is strongly associated with an increased risk of type 2 diabetes, cardiovascular diseases, and premature mortality. Direct and indirect costs associated with these complications represent a significant burden for Latin American health systems, which already face considerable budgetary constraints (5).

The marked difference in prevalence between sexes, with significantly higher involvement in women, suggests the need for gender-specific public health policies. This finding is particularly relevant considering that Latin American women are frequently the cornerstone of family economics and play a fundamental role in household dietary decisions (84).

The need for standardization of diagnostic criteria is urgent. Variability in measurement methods and cut-off points hinders comparison between studies and could lead to misclassification of cardiovascular risk in the population. It is imperative to develop regional consensuses that establish specific cut-off points for the Latin American population based on local evidence and consider the region’s ethnic and anthropometric characteristics. This would allow better identification of at-risk individuals and more efficient allocation of prevention resources.

The disease burden associated with AO in Latin America is substantial and growing. According to Pan American Health Organization estimates, cardiometabolic diseases associated with AO represent the leading cause of premature death in the region. Direct healthcare costs attributable to this condition are estimated at US$ 20 billion annually, without considering indirect costs from productivity loss and disability-adjusted life years.

## Limitations and strengths

This systematic review presents both significant strengths and limitations to consider. Notable strengths include: the broad temporal period analyzed (2005-2024), the inclusion of multiple Latin American countries, the large cumulative sample size (over 280,000 participants), and the rigorous assessment of risk of bias using validated tools. However, limitations include: considerable heterogeneity between studies (I² > 99%), variability in WC measurement methods, with only 59% of studies following standardized protocols, lack of representativeness of some Latin American countries (for example, Bolivia, Paraguay, and Uruguay are not represented), and the impossibility of conducting stratified analyses by socioeconomic level or rural/urban area due to lack of consistent data.

## Conclusions and recommendations

This SR demonstrates a high prevalence of AO in Latin America, with marked differences according to diagnostic criteria used (40% according to ATP III and 62% according to IDF) and significantly higher involvement in women. The findings question the validity of current cut-off points and suggest the urgent need to develop specific criteria for the Latin American population based on clinically relevant outcomes, not just anthropometric correlations.

Given these results, we recommend: 1) establishing a Latin American consensus to standardize WC measurement methods; 2) developing prospective studies that evaluate the association between different cut-off points and cardiovascular outcomes in Latin populations; 3) implementing gender-differentiated public health policies, considering the higher prevalence in women; and 4) strengthening epidemiological surveillance systems to monitor temporal trends of AO in the region. The magnitude of the identified problem requires urgent and coordinated actions at the regional level to prevent and control this growing threat to Latin American public health.

## Data Availability

The raw data supporting the conclusions of this article will be made available by the authors, without undue reservation.
