# Supplementary material for: Abdominal obesity prevalence in Latin America: a systematic review and meta-analysis comparing ATP III and IDF criteria
Source: Front Endocrinol (Lausanne). 2025 Jun 17;16:1562060. doi: 10.3389/fendo.2025.1562060 (PMC12208830; doi:10.3389/fendo.2025.1562060)
Supplement: Supplementary file 1 [file DataSheet1.pdf]

Supplementary file 1

Search strategy in PUBMED	
#1	obesity[MeSH] OR obesity[tiab] OR obese[tiab] OR "abdominal obesity"[tiab] OR "central adiposity"[tiab] OR "central adiposity"[tiab]
#2	"waist circumference"[MeSH] OR "waist circumference"[tiab] OR WC[tiab] OR "abdominal circumference"[tiab] OR "waist-to-height ratio"[tiab] OR WHtR[tiab] OR "waist-height ratio"[tiab] OR "waist-to-stature ratio"[tiab] OR "Metabolic Syndrome"[MeSH] OR "metabolic syndrome"[tiab] OR "syndrome X"[tiab] OR "insulin resistance syndrome"[tiab]
#3	"Antigua and Barbuda"[TIAB] OR "Antigua y Barbuda"[TIAB] OR "Argentina"[TIAB] OR "Bahamas"[TIAB] OR "Barbados"[TIAB] OR "Belize"[TIAB] OR "Bolivia"[TIAB] OR "Brazil"[TIAB] OR "Brasil"[TIAB] OR "United States Virgin Islands"[TIAB] OR "British Virgin Islands"[TIAB] OR "Chile"[TIAB] OR "Colombia"[TIAB] OR "Costa Rica"[TIAB] OR "Cuba"[TIAB] OR "Dominica"[TIAB] OR "Dominican Republic"[TIAB] OR "República Dominicana"[TIAB] OR "Ecuador"[TIAB] OR "El Salvador"[TIAB] OR "Grenada"[TIAB] OR "Granada"[TIAB] OR "Guatemala"[TIAB] OR "Guyana"[TIAB] OR "Haiti"[TIAB] OR "Haïti"[TIAB] OR "Honduras"[TIAB] OR "Jamaica"[TIAB] OR "Mexico"[TIAB] OR "México"[TIAB] OR "Nicaragua"[TIAB] OR "Panama"[TIAB] OR "Panamá"[TIAB] OR "Paraguay"[TIAB] OR "Peru"[TIAB] OR "Perú"[TIAB] OR "Puerto Rico"[TIAB] OR "Saint Kitts and Nevis"[TIAB] OR "San Cristóbal y Nieves"[TIAB] OR "San Cristobal y Nieves"[TIAB] OR "Saint Lucia"[TIAB] OR "Santa Lucía"[TIAB] OR "Saint Vincent and the Grenadines"[TIAB] OR "San Vicente y las Granadinas"[TIAB] OR "Suriname"[TIAB] OR "Surinam"[TIAB] OR "Trinidad and Tobago"[TIAB] OR "Trinidad y Tobago"[TIAB] OR "West Indies"[TIAB] OR "Indias occidentales"[TIAB] OR "Uruguay"[TIAB] OR "Venezuela"[TIAB] OR "Latin America"[TIAB] OR "latinoamérica"[TIAB] OR "latin amer*"[TIAB] OR "South America"[TIAB] OR "south amer*"[TIAB] OR "Central America"[TIAB] OR "central amer*"[TIAB]
#4	#1 AND #2 AND #3
Search strategy in SCOPUS	
#1	TITLE-ABS-KEY (obesity OR obese OR overweight* OR "body weight" OR adipos* OR "body fat" OR "fat mass" OR "abdominal obesity" OR "central obesity" OR "visceral obesity" OR "abdominal adiposity" OR "central adiposity" OR "visceral adiposity" OR "body mass index" OR BMI OR "quetelet index")
#2	TITLE-ABS-KEY ("waist circumference" OR "waist circumferences" OR WC OR "abdominal circumference" OR "abdominal circumferences" OR "waist-to-height ratio" OR WHtR OR "waist-height ratio" OR "waist-to-stature ratio" OR WSR OR "waist-hip ratio" OR WHR OR "abdominal obesity" OR "central obesity" OR "visceral obesity" OR "metabolic syndrome" OR "syndrome X" OR "insulin resistance syndrome" OR "cardiometabolic risk" OR "cardiometabolic syndrome" OR "dysmetabolic syndrome")
#3	("Antigua and Barbuda" OR "Antigua y Barbuda" OR Antigua OR Barbuda OR Argentina OR Argentin* OR Bahamas OR Barbados OR Belize OR Belice OR Bolivia OR Bolivian* OR Brazil OR Brasil OR Brazilian* OR "Virgin Islands" OR Chile OR Chilean* OR Colombia OR Colombian* OR "Costa Rica" OR "Costa Rican" OR Cuba OR Cuban* OR Dominica OR "Dominican Republic" OR "República Dominicana" OR Dominican* OR Ecuador OR Ecuadorian* OR "El Salvador" OR Salvadoran* OR Grenada OR Granada OR Grenadian* OR Guatemala OR Guatemalan* OR Guyana OR Guyanese OR Haiti OR Haïti OR Haitian* OR Honduras OR Honduran* OR Jamaica OR Jamaican* OR Mexico OR México OR Mexican* OR Nicaragua OR Nicaraguan* OR Panama OR Panamá OR Panamanian* OR Paraguay OR Paraguayan* OR Peru OR Perú OR Peruvian* OR "Puerto Rico" OR "Puerto Rican" OR "Saint Kitts and Nevis" OR "San Cristóbal y Nieves" OR "Saint Lucia" OR "Santa Lucía" OR "Saint Vincent and the Grenadines" OR "San Vicente y las Granadinas" OR Suriname OR Surinam OR Surinamese OR "Trinidad and Tobago" OR "Trinidad y Tobago" OR Trinidadian* OR "West Indies" OR "Indias occidentales" OR Uruguay OR Uruguayan* OR Venezuela OR Venezuelan* OR "Latin America*" OR latinoamérica OR "South America*" OR "Central America*" OR Caribbean OR Caribe*)

#4	#1 AND #2 AND #3
Search strategy in Web of Science	
#1	TS=(obesity OR obese OR overweight* OR "body weight" OR adipos* OR "body fat" OR "fat mass" OR "abdominal obesity" OR "central obesity" OR "visceral obesity" OR "abdominal adiposity" OR "central adiposity" OR "visceral adiposity" OR "body mass index" OR BMI OR "quetelet index")
#2	TS=("waist circumference" OR "waist circumferences" OR WC OR "abdominal circumference" OR "abdominal circumferences" OR "waist-to-height ratio" OR WHtR OR "waist-height ratio" OR "waist-to-stature ratio" OR WSR OR "waist-hip ratio" OR WHR OR "abdominal obesity" OR "central obesity" OR "visceral obesity" OR "metabolic syndrome" OR "syndrome X" OR "insulin resistance syndrome" OR "cardiometabolic risk" OR "cardiometabolic syndrome" OR "dysmetabolic syndrome")
#3	TS=("Antigua and Barbuda" OR "Antigua y Barbuda" OR Antigua OR Barbuda OR Argentina OR Argentin* OR Bahamas OR Barbados OR Belize OR Belice OR Bolivia OR Bolivian* OR Brazil OR Brasil OR Brazilian* OR "Virgin Islands" OR Chile OR Chilean* OR Colombia OR Colombian* OR "Costa Rica" OR "Costa Rican" OR Cuba OR Cuban* OR Dominica OR "Dominican Republic" OR "República Dominicana" OR Dominican* OR Ecuador OR Ecuadorian* OR "El Salvador" OR Salvadoran* OR Grenada OR Granada OR Grenadian* OR Guatemala OR Guatemalan* OR Guyana OR Guyanese OR Haiti OR Haïti OR Haitian* OR Honduras OR Honduran* OR Jamaica OR Jamaican* OR Mexico OR México OR Mexican* OR Nicaragua OR Nicaraguan* OR Panama OR Panamá OR Panamanian* OR Paraguay OR Paraguayan* OR Peru OR Perú OR Peruvian* OR "Puerto Rico" OR "Puerto Rican" OR "Saint Kitts and Nevis" OR "San Cristóbal y Nieves" OR "Saint Lucia" OR "Santa Lucía" OR "Saint Vincent and the Grenadines" OR "San Vicente y las Granadinas" OR Suriname OR Surinam OR Surinamese OR "Trinidad and Tobago" OR "Trinidad y Tobago" OR Trinidadian* OR "West Indies" OR "Indias occidentales" OR Uruguay OR Uruguayan* OR Venezuela OR Venezuelan* OR "Latin America*" OR latinoamérica OR "South America*" OR "Central America*" OR Caribbean OR Caribe*)
#4	#1 AND #2 AND #3
Search strategy in EMBASE	
#1	OR 'body weight'/exp OR 'body weight':ti,ab,kw OR 'adiposity'/exp OR adipos*:ti,ab,kw OR 'body fat':ti,ab,kw OR 'fat mass':ti,ab,kw OR 'abdominal obesity'/exp OR 'abdominal obesity':ti,ab,kw OR 'central obesity':ti,ab,kw OR 'visceral obesity':ti,ab,kw OR 'central adiposity':ti,ab,kw OR 'body mass'/exp OR 'body mass index':ti,ab,kw OR 'bmi':ti,ab,kw OR 'quetelet index':ti,ab,kw)
#2	('waist circumference'/exp OR 'waist circumference':ti,ab,kw OR 'wc':ti,ab,kw OR 'abdominal circumference':ti,ab,kw OR 'waist to height ratio'/exp OR 'waist-to-height ratio':ti,ab,kw OR 'whtr':ti,ab,kw OR 'waist height ratio':ti,ab,kw OR 'waist to stature ratio':ti,ab,kw OR 'wsr':ti,ab,kw OR 'waist hip ratio'/exp OR 'waist-hip ratio':ti,ab,kw OR 'whr':ti,ab,kw OR 'metabolic syndrome X'/exp OR 'metabolic syndrome':ti,ab,kw OR 'syndrome x':ti,ab,kw OR 'insulin resistance syndrome':ti,ab,kw OR 'cardiometabolic risk'/exp OR 'cardiometabolic risk':ti,ab,kw OR 'cardiometabolic syndrome':ti,ab,kw OR 'dysmetabolic syndrome':ti,ab,kw)
#3	('Antigua and Barbuda'/exp OR 'Argentina'/exp OR 'Bahamas'/exp OR 'Barbados'/exp OR 'Belize'/exp OR 'Bolivia'/exp OR 'Brazil'/exp OR 'Virgin Islands'/exp OR 'Chile'/exp OR 'Colombia'/exp OR 'Costa Rica'/exp OR 'Cuba'/exp OR 'Dominica'/exp OR 'Dominican Republic'/exp OR 'Ecuador'/exp OR 'El Salvador'/exp OR 'Grenada'/exp OR 'Guatemala'/exp OR 'Guyana'/exp OR 'Haiti'/exp OR 'Honduras'/exp OR 'Jamaica'/exp OR 'Mexico'/exp OR 'Nicaragua'/exp OR 'Panama'/exp OR 'Paraguay'/exp OR 'Peru'/exp OR 'Puerto Rico'/exp OR 'Saint Kitts and Nevis'/exp OR 'Saint Lucia'/exp OR 'Saint Vincent and the Grenadines'/exp OR 'Suriname'/exp OR 'Trinidad and Tobago'/exp OR 'Uruguay'/exp OR 'Venezuela'/exp OR 'Latin America'/exp OR 'South America'/exp OR 'Central America'/exp OR 'Caribbean'/exp OR (('Antigua and Barbuda' OR 'Antigua y Barbuda' OR Antigua OR Barbuda OR Argentina OR Argentin* OR Bahamas OR Barbados OR Belize OR Belice OR Bolivia OR Bolivian* OR Brazil OR Brasil OR Brazilian* OR 'Virgin Islands' OR Chile OR Chilean* OR Colombia OR Colombian* OR 'Costa Rica' OR 'Costa Rican' OR Cuba OR Cuban* OR Dominica OR

	'Dominican Republic' OR 'República Dominicana' OR Dominican* OR Ecuador OR Ecuadorian* OR 'El Salvador' OR Salvadoran* OR Grenada OR Granada OR Grenadian* OR Guatemala OR Guatemalan* OR Guyana OR Guyanese OR Haiti OR Haïti OR Haitian* OR Honduras OR Honduran* OR Jamaica OR Jamaican* OR Mexico OR México OR Mexican* OR Nicaragua OR Nicaraguan* OR Panama OR Panamá OR Panamanian* OR Paraguay OR Paraguayan* OR Peru OR Perú OR Peruvian* OR 'Puerto Rico' OR 'Puerto Rican' OR 'Saint Kitts and Nevis' OR 'San Cristóbal y Nieves' OR 'Saint Lucia' OR 'Santa Lucía' OR 'Saint Vincent and the Grenadines' OR 'San Vicente y las Granadinas' OR Suriname OR Surinam OR Surinamese OR 'Trinidad and Tobago' OR 'Trinidad y Tobago' OR Trinidadian* OR 'West Indies' OR 'Indias occidentales' OR Uruguay OR Uruguayan* OR Venezuela OR Venezuelan* OR 'Latin America*' OR latinoamérica OR 'South America*' OR 'Central America*' OR Caribbean OR Caribe*):ti,ab,kw))
#4	#1 AND #2 AND #3
