# Supplementary material for: Abdominal obesity prevalence in Latin America: a systematic review and meta-analysis comparing ATP III and IDF criteria
Source: Front Endocrinol (Lausanne). 2025 Jun 17;16:1562060. doi: 10.3389/fendo.2025.1562060 (PMC12208830; doi:10.3389/fendo.2025.1562060)
Supplement: Supplementary file 2 [file DataSheet2.pdf]

Supplementary material 2. Summary of selected studies

First Author, Year	Country	Study Type	Inclusion Criteria	Exclusion Criteria	Sample Size	Sex (% Female)	Age (Mean)	WC Measurement Method	WC Cutoff	Database (Year of Collection)
Florez (2005) (11)	Venezuela	Cross-sectional	Men and women aged 20 years or older residing in the state of Zulia, Venezuela.	-	3,108	69.6	-	Abdominal circumference was measured at the umbilical level using a non-stretchable measuring tape.	≥88 cm for women; ≥102 cm for men.	Collected for the study.
Marcopito (2005) (12)	Brazil	Cross-sectional	Individuals aged 15–59 years residing in São Paulo.	Pregnant or postpartum women.	2,103	53.5	-	Measured using a measuring tape at the waistline.	≥88 cm for women; ≥102 cm for men.	Collected for the study.
Anselmo (2006) (13)	Brazil	Cross-sectional	Adults aged 20–69 years residing in urban areas of Pelotas, Brazil.	-	1,935	56.7	42	Abdominal circumference was measured with a non-stretchable measuring tape at the midpoint between the last rib and the iliac crest.	≥88 cm for women; ≥102 cm for men.	Collected for the study.
Mohanna (2006) (14)	Peru	Cross-sectional	Native residents of San Pedro de Cajas aged 30 years or older, living in the area for at least 10 years.	Participants with conditions such as ascites, hypothyroidism, cirrhosis, chronic kidney disease, pregnant women, and those undergoing treatment with corticosteroids or beta-blockers.	102	62.7	47.6	Abdominal circumference was measured using a non-stretchable tape following international standards at the midpoint between the last rib and the iliac crest.	≥88 cm for women; ≥102 cm for men.	Collected for the study.

Medina (2007) [15]	Peru	Cross-sectional	Adults aged 20 years or older residing in Arequipa, Peru.	-	1,878	53.8	48.5 women; 49.6 men	Abdominal circumference was measured at the umbilical level using a non-stretchable measuring tape while participants stood with feet together, arms at their sides, and breathing normally.	≥88 cm for women; ≥102 cm for men.	Collected for the study.
Perozzo (2008) [16]	Brazil	Cross-sectional	Women aged 20–60 years residing in the urban area of São Leopoldo, Rio Grande do Sul.	-	1,026	100	-	Abdominal circumference was measured using a non-stretchable tape at the smallest diameter between the last rib and the iliac crest.	≥88 cm for women.	Collected for the study.
Sá (2009) [17]	Brazil	Cross-sectional	Individuals aged 20 years or older residing in Salvador, Bahia, with complete data on pain and anthropometric measurements.	Pregnant women.	2,297	55.4	40.9	Abdominal circumference was measured using a non-stretchable metallic tape at the narrowest point of the torso between the chest and the hip.	>88 cm for women; >83 cm for men.	Chronic Disease Monitoring Project of Salvador's Population (MONIT).
Cárdenas (2009) [18]	Peru	Cross-sectional	Individuals aged 20 years or older with complete anthropometric measurements and biochemical	-	4,053	50.3	-	Abdominal circumference was measured using a measuring tape and recorded at the midpoint between	≥88 cm for women; ≥102 cm for men.	National Survey of Nutritional, Biochemical, Socioeconomic, and Cultural Indicators.

			analyses, residing in Peru.					the last rib and the iliac crest.		
Parra (2009) [19]	Colombia	Cross-sectional	Adults aged 18–64 years residing in urban areas of Colombia.	-	47,833	62.5	36.5	Abdominal circumference was measured using a measuring tape placed parallel to the floor at the midpoint between the iliac crest and the last rib after a relaxed exhalation.	≥90 cm for men; ≥80 cm for women.	Colombia Demographic and Health Survey (2005).
Rojas (2010) [20]	Mexico	Cross-sectional	Mexican adults aged 20 years or older residing in urban and rural areas.	-	6,021	-	-	Abdominal circumference was measured using a non-stretchable tape at the narrowest point between the last rib and the iliac crest.	ATPIII: ≥88 cm for women; ≥102 cm for men. IDF: ≥80 cm for women; ≥94 cm for men.	National Health and Nutrition Survey 2006 (ENSANUT 2006).
Ferreira (2010) [21]	Brazil	Cross-sectional	Adults aged 20–59 years residing in the six most populous municipalities of Maranhão, Brazil.	Participants with incomplete or inconsistent measurement responses.	1,005	60.9	-	A non-flexible measuring tape was used to measure abdominal circumference at the midpoint between the last rib and the iliac crest during exhalation.	≥90 cm for men; ≥80 cm for women.	Collected for the study.
Patiño (2011) [22]	Colombia	Cross-sectional	Men and women aged 25–50 years	Individuals with sensory, cognitive, or motor	357	60.2	-	Abdominal circumference was	≥90 cm for men;	Collected for the study.

			residing for at least 3 years in the urban area of Santa Rosa de Osos, Antioquia, Colombia.	disabilities, psychiatric conditions, established cardiovascular diseases, and pregnant women.				measured using a tape at the narrowest part between the chest and the hips.	≥80 cm for women.	
Marcal (2011) [22]	Brazil	Cross-sectional	Rural residents of Minas Gerais aged 18 years or older.	Pregnant women, individuals with diabetes, those with CRP >10 mg/L (indicative of acute infection or inflammation), and individuals with physical disabilities preventing anthropometric measurements.	534	49.4	-	Abdominal circumference was measured using a non-elastic tape placed at the midpoint between the last costal arch and the iliac crest during normal expiration.	≥88 cm for women; ≥102 cm for men.	Collected for the study.
Guilherme (2011) [24]	Brazil	Cross-sectional	Women aged 20–60 years registered in the Family Health Program.	Women with physical disabilities or pregnant at the time of the study.	632	100	42.6	Measured with a non-stretchable tape at the midpoint between the iliac crest and the lower rib.	≥88 cm for women.	Collected for the study.
Zarbato (2011) [25]	Brazil	Cross-sectional	Adults aged 20–59 years, both sexes, residing in the urban area of Lages, SC.	Pregnant individuals, amputees, bedridden participants, those with orthopedic devices, or psychiatric disabilities.	2,022	52.35	-	Measured at the natural waistline (narrowest circumference) using a non-elastic tape.	≥88 cm for women; ≥102 cm for men.	Collected for the study.
Bermúdez (2012) [26]	Venezuela	Cross-sectional	Adults aged 18 years or older residing in Maracaibo, randomly selected.	-	2,108	53.09	38.68	NIH: Measurement was taken at the midpoint between the lower edge of the rib cage and the iliac crest at the end of expiration with	ATPIII: ≥88 cm for women; ≥102 cm for men. IDF: ≥80 cm for	Metabolic Syndrome Prevalence Database of Maracaibo (MMPS).

								participants standing.	women; ≥94 cm for men.	
Da Silva (2012) [27]	Brazil	Cross-sectional	Adults aged 20 years or older residing in the urban area of Pelotas, Rio Grande do Sul.	Institutionalized individuals, pregnant women, or those who had given birth within the past six months, and individuals with disabilities preventing anthropometric measurements.	2,448	58.3	-	Abdominal circumference was measured using a non-flexible tape directly on the skin at the narrowest point between the chest and the hips.	≥88 cm for women; ≥102 cm for men.	Collected for the study.
Pérez (2012) [28]	Puerto Rico	Cross-sectional	Adults aged 21–79 years residing in the metropolitan area of San Juan, Puerto Rico, identifying as Puerto Rican.	Participants with incomplete data to define metabolic syndrome.	858	65.6	49.4	Abdominal circumference was measured using a measuring tape at the highest point of the iliac crest during minimal respiration.	≥88 cm for women; ≥102 cm for men.	Collected for the study.
Loret (2012) [29]	Peru	Cross-sectional	Permanent residents of rural and urban areas of Lima and Ayacucho aged 30 years or older.	Pregnant women and individuals unable to understand or provide informed consent.	983	53	48	A non-stretchable tape was used to measure abdominal circumference at the midpoint between the lower rib and the iliac crest.	≥88 cm for women; ≥102 cm for men.	PERU MIGRANT Study.

Sabino (2013) [30]	Brazil	Cross-sectional	Adults aged 25–59 years residing in urban and rural areas of Pernambuco State, Brazil.	Individuals with physical limitations preventing anthropometric measurements, pregnant women, and women who gave birth in the previous six months.	1,580	58	-	Abdominal circumference was measured using a 200 cm non-stretchable tape at the midpoint between the last rib and the iliac crest, following WHO protocol.	≥80 cm for women; ≥94 cm for men.	Collected for the study.
Bresciani (2013) [31]	Brazil	Cross-sectional	Bank employees aged 20–64 years in Vitória/ES, Brazil.	Participants who did not undergo biochemical tests.	501	49.1	-	Abdominal circumference was measured at the lowest point between the last rib and the iliac crest using a measuring tape.	≥90 cm for men; ≥80 cm for women.	Collected for the study.
Jiménez (2013) [32]	Mexico	Cross-sectional	Adults aged 18 years or older residing in four neighborhoods of Tijuana, Baja California.	-	322	70	39	Abdominal circumference was measured using a non-stretchable tape at the narrowest point between the iliac crest and the lower edge of the ribs.	≥88 cm for women; ≥102 cm for men.	Collected for the study.
Rodrigues (2013) [33]	Brazil	Cross-sectional	Non-pregnant women aged 18–49 years with available WC measurements.	Pregnant women.	13,262	100	-	Measured twice at the midpoint between the last rib and the iliac crest with a non-elastic tape; the average value was recorded.	≥80 cm for women.	National Demographic and Health Survey (PNDS 2006–2007).

Salas (2014) [34]	Mexico	Cross-sectional	Adults aged 16 years or older participating in the Nuevo León State Nutrition and Health Survey (2011/2012).	-	1,200	51.1	-	Abdominal circumference was measured using a non-stretchable tape at the midpoint between the lower edge of the ribs and the iliac crest while participants stood upright.	≥90 cm for men; ≥80 cm for women.	Nuevo León State Nutrition and Health Survey 2011/2012.
Ibañez (2014) [35]	Chile	Cohort	Mapuche individuals aged 18 years or older residing in rural or urban areas of Biobío and Los Ríos regions.	Individuals with non-Mapuche phenotypic characteristics (e.g., white skin, blonde hair), residing in the area for less than 2 years, mental health issues, or pregnancy.	1,077	59.5	-	Abdominal circumference was measured using a non-stretchable tape at the midpoint between the last rib and the iliac crest.	ATPIII: ≥88 cm for women; ≥102 cm for men. IDF: ≥80 cm for women; ≥94 cm for men.	Collected for the study.
Costa (2014) [36]	Brazil	Cross-sectional	Professors at the Federal University of Viçosa (UFV), regardless of sex, ethnicity, or age.	-	145	28.97	46.9 men; 43.2 women	Abdominal circumference was measured using a non-stretchable tape at the umbilical level.	≥88 cm for women; ≥102 cm for men.	Collected for the study.
Muñoz (2014) [37]	Puerto Rico	Cross-sectional	Adults aged 70 years or older residing in the metropolitan area of San Juan.	Individuals with medical conditions contraindicating periodontal probing, or a history of myocardial infarction or cancer.	147	67.3	77.7	Measured with a measuring tape above the iliac crest according to CDC-AAP criteria.	≥88 cm for women; ≥102 cm for men.	Puerto Rican Elderly Dental Health Study (PREDHS).
Alvarez (2014) [38]	Colombia	Cross-sectional	Adults aged 18–64 years residing in urban and rural	-	5,556	61.7	40	Measured with a non-retractable tape at the midpoint	≥80 cm for women;	Medellín Food and Nutrition

			households in Medellín.					between the last rib and the iliac crest.	≥94 cm for men.	Profile Survey 2010.
Boing (2015) [39]	Brazil	Cross-sectional	Adults aged 20–59 years residing in Florianópolis, Brazil, with complete anthropometric measurements.	Individuals with amputations, bedridden individuals, or those unable to complete the questionnaire.	1,720	55.5	37	Abdominal circumference was measured with a non-elastic tape at the narrowest point of the trunk or at the midpoint between the iliac crest and the last rib.	≥88 cm for women; ≥102 cm for men.	Collected for the study.
Orellana (2015) [40]	Guatemala	Cross-sectional	Men and women aged 18 years or older, born and residing in Sololá Department, Guatemala.	Participants who refused to provide informed consent.	1,104	50	37.1	Abdominal circumference was measured at the iliac crest level using a non-elastic tape, with participants in a standing position.	≥80 cm for women; ≥94 cm for men.	Collected for the study.
Dos Santos Costa (2016) [41]	Brazil	Cross-sectional	Individuals aged 60 years or older residing in urban areas of Pelotas, RS.	Individuals unable to maintain an upright position, or with prosthetics, casts, or lower limb amputations.	1,446	63	70.7	Abdominal circumference was measured with a non-stretchable tape directly on the skin at the narrowest point of the trunk or, if not possible, at the midpoint between the last rib and the iliac crest.	≥88 cm for women; ≥102 cm for men.	Collected for the study.
Kumar (2016) [42]	Mexico	Cross-sectional	Mexican adults aged 50 years or older and their	Participants with incomplete HbA1c information or those who had previously reported	2,012	53.3	61.9	Not specified.	≥88 cm for women;	Mexican Health and Aging Study (MHAS).

			spouses or partners.	diabetes in earlier waves of the study.					≥102 cm for men.	
Barengo (2016) [43]	Colombia	Cross-sectional	Individuals aged 18–74 years without a prior diabetes diagnosis.	Diagnosed diabetes, pregnancy, cancer, use of corticosteroids.	2,060	62	47.2	Measured at the midpoint between the last rib and the iliac crest.	≥94 cm for men; ≥90 cm for women.	Data from Mutual SER EPSS (health insurance system).
Rosada (2016) [44]	Brazil	Cross-sectional	Individuals aged 60 years or older living independently.	Participants who did not attend all evaluations.	489	66.5	-	Measured with a measuring tape following World Health Organization criteria.	≥94 cm for men; ≥80 cm for women.	EELO Project.
Pires (2017) [45]	Brazil	Longitudinal	Individuals aged 60 years or older, independent in daily activities, and participants in the second phase of the SABE study in 2006.	Participants with incomplete anthropometric data or who developed difficulties in daily activities before 2006.	1,109	57	69.2	Abdominal circumference was measured using a non-stretchable tape at the midpoint between the last rib and the iliac crest, at the end of expiration, with the participant standing.	≥88 cm for women; ≥102 cm for men.	SABE - São Paulo Study.
Orces (2017) [46]	Ecuador	Cross-sectional	Individuals aged 60 years or older residing in the Andean and coastal regions of Ecuador.	-	2,298	54.7	71.6	Abdominal circumference was measured using a non-stretchable tape at the midpoint between the last rib and the iliac crest.	≥90 cm for men; ≥80 cm for women.	SABE (National Health, Wellbeing, and Aging Survey).

Bernabé (2017) [47]	Peru	Cohort	Participants of the PERU MIGRANT cohort with complete blood pressure information.	Individuals with hypertension diagnosed at baseline.	988	52.9	47.9	Measured three times by trained field workers using standardized techniques.	≥90 cm for men; ≥80 cm for women.	PERU MIGRANT Study Data.
Mulatinho (2018) [48]	Brazil	Cross-sectional	Adults aged 24–59 years who were permanent residents of Fernando de Noronha with over 10 years of residence or were born in the archipelago.	-	375	68.5	43.81	A non-elastic tape was used, placed at the midpoint between the lower edge of the rib cage and the iliac crest, with a precision of 0.1 cm.	≥90 cm for men; ≥80 cm for women.	Collected for the study.
Pudla (2018) [49]	Brazil	Cross-sectional	Adults aged 22–63 years residing in Florianópolis, Brazil, who participated in the follow-up of the EpiFloripa cohort in 2012.	Institutionalized adults or those with physical or mental disabilities preventing questionnaire responses.	1,222	57.3	44.9	WC was measured using a non-stretchable tape at the narrowest part of the torso, or at the midpoint between the iliac crest and the last rib for those without a visible waist.	≥88 cm for women; ≥102 cm for men.	EpiFloripa Adult Cohort Study.
Vitorino (2018) [50]	Brazil	Cross-sectional	Users of the Unified Health System (SUS) in Brazil aged 20 years or older.	Pregnant women or individuals with cognitive conditions interfering with questionnaire responses.	384	80.5	42.5	Measured between the last rib and the iliac crest.	≥90 cm for men; ≥80 cm for women.	Collected for the study.
Carrillo-Larco (2018) [51]	Peru	Cohort	Individuals aged 35 years or older.	Pregnant women unable to provide informed consent or individuals unable to complete the questionnaires.	3,217	51.6	55.7 (SD 12.7)	Not specified.	≥90 cm for men; ≥80 cm for women.	COHORT CRONICAS.

Martins-Silva (2019) [52]	Brazil	Cross-sectional	Adults aged 18 years or older with complete anthropometric data.	Pregnant women or those suspected of being pregnant at the time of the survey.	59,226	56.23	-	WHO: Participants were standing, and the measurement was taken after a regular exhalation. A non-stretchable tape with 0.1 cm precision was used, placed horizontally at the midpoint between the lowest rib and the iliac crest.	≥88 cm for women; ≥102 cm for men.	National Health Survey (PNS) 2013.
Petermann (2019) [53]	Chile	Cross-sectional	Chilean adults aged 18 years or older with complete data on physical activity and anthropometric measurements.	Pregnant women and individuals unable to provide informed consent.	5,157	59.3	-	WC was measured using a non-stretchable tape at the midpoint between the lower edge of the rib and the iliac crest, with the participant standing.	≥88 cm for women; ≥102 cm for men.	Chile National Health Survey (ENS) 2009/10.
Pajuelo (2019) [54]	Peru	Cross-sectional	Adults aged 20 years or older residing in Peru, excluding pregnant women.	Pregnant women, incomplete data.	20,489	51.6	-	Measured using a tape placed between the last rib and the iliac crest.	ATPIII: ≥88 cm for women; ≥102 cm for men. IDF: ≥80 cm for women; ≥94 cm for men.	National Household Survey (ENAHO) 2013.

Scherlowski (2020) [55]	Brazil	Cross-sectional	Adults aged 20–59 years residing in Florianópolis, Brazil.	Amputees, bedridden individuals, hospitalized patients, or those with severe mental or physical illnesses compromising anthropometric measurements.	605	-	39.6	Abdominal circumference was measured using a non-elastic anthropometric tape at the midpoint between the iliac crest and the lower edge of the costal arch.	≥90 cm for men; ≥80 cm for women.	EpiFloripa Adults Cohort Study.
Higuita (2020) [56]	Colombia	Cross-sectional	Active participation in the chronic disease control program during 2018, with at least two follow-ups.	Individuals under 18, fewer than two follow-up visits, or logically inconsistent clinical records.	68,288	72.2	-	Measured according to the consensus of the Latin American Diabetes Association (ALAD).	≥80 cm for women; ≥94 cm for men.	Data from Metrosalud's chronic disease control program, Medellín.
Barranco (2020) [57]	Colombia	Cross-sectional	Adults aged 60 years or older in Colombia.	-	1,637	60.7	70.5	Measured with a measuring tape.	≥90 cm for men; ≥80 cm for women.	SABE Colombia Survey 2015.
Santana (2020) [58]	Brazil	Cross-sectional	Workers aged 18 years or older who had been employed for more than one year.	Workers on probation, interns, and pregnant women.	676	42.7	-	Measured at the midpoint between the last rib and the iliac crest.	≥80 cm for women; ≥94 cm for men.	Collected for the study.
Ortiz (2021) [59]	Mexico	Cross-sectional	Mexican adults aged 20 years or older affiliated with the ISSSTE.	Participants with incomplete data on key variables such as metabolic syndrome, diabetes, or hypertension.	4,595	66.8	50	Abdominal circumference was measured using a fiberglass tape at the smallest point between the ribs and iliac crests during minimal respiration.	≥90 cm for men; ≥80 cm for women.	Collected for the study.

De Sousa (2021) [60]	Brazil	Cross-sectional	Self-declared quilombola individuals aged 18 years or older residing in selected communities.	Individuals with mental or cognitive impairments reported by family or healthcare teams, or those who did not authorize abdominal circumference measurement.	1,025	60.9	-	Measured using a 150 cm non-stretchable tape at the midpoint between the 10th rib and the upper edge of the iliac crest.	≥90 cm for men; ≥80 cm for women.	Collected for the study.
Bello (2021) [61]	Mexico	Cohort	Adults aged 25–69 years without type 2 diabetes, hypertension, or significant cardiovascular comorbidities.	Participants with type 2 diabetes, heart disease, cerebrovascular, renal, or liver diseases.	6,144	67.9	40	Measured with a tape measure following ATP-III and IDF protocols.	≥88 cm for women; ≥102 cm for men.	Collected for the study.
Farro (2021) [62]	Peru	Cross-sectional	Adults aged 18 years or older with waist circumference measurements in ENDES 2018–2019.	Pregnant women, incomplete data.	62,138	56.9	-	Measured using a tape measure according to IDF criteria.	≥90 cm for men; ≥80 cm for women.	Peru National Demographic and Family Health Survey (ENDES) 2018–2019.
Aparco (2022) [63]	Peru	Cross-sectional	Adults aged 18–59 years who were fasting for at least 9 hours and no more than 12 hours.	Adults aged 18–59 years not on the identification list, pregnant/postpartum women, adults undergoing treatments affecting glucose or lipid profiles, those not fasting, gastrointestinal conditions affecting food intake, and genetic conditions or malformations affecting anthropometric techniques.	1,047	57.6	21.4	Not specified.	≥90 cm for men; ≥80 cm for women.	VIANEV.

Campos-Nonato (2022) [64]	Mexico	Cross-sectional	Adults aged 20 years or older with complete WC data included in the analysis.	Participants with implausible height <130 cm or WC <50 cm or >200 cm, and pregnant women.	8,563	52.8	-	WC was measured.	≥90 cm for men; ≥80 cm for women.	ENSANUT.
Rodrigues (2023) [65]	Brazil	Cross-sectional	Women aged 30–80 years without a cancer diagnosis.	Individuals with orthopedic or neurological problems, or cognitive impairment.	150	100	54.07	Measured with a measuring tape.	≥80 cm for women.	Collected for the study.
Do Nascimento (2023) [66]	Brazil	Cross-sectional	Adults aged 60 years or older residing in Macapá.	Individuals with neurological sequelae, hospitalized patients, or those with cognitive impairment.	382	-	70.03	Measured with a tape between the iliac crest and the last rib.	≥88 cm for women; ≥102 cm for men.	Collected for the study.
Tavares (2023) [67]	Brazil	Cross-sectional	Adults aged 50 years or older residing in urban and rural areas of Brazil.	Participants with incomplete data or residing in institutions.	9,949	59.3	-	Measured with a tape measure.	≥80 cm for women; ≥94 cm for men.	Brazilian Longitudinal Study of Aging (ELSI-Brasil).
Natal (2023) [68]	Brazil	Cross-sectional	Adults aged 35–74 years, public employees, without recent changes in antihypertensive medication.	Pregnant women, incomplete data, or residents outside the metropolitan study areas.	812	50	51	Measured at the midpoint between the last rib and the iliac crest.	≥90 cm for men; ≥80 cm for women.	Brazilian Longitudinal Study of Adult Health (ELSA-Brasil).
Alvarez (2024) [69]	Mexico	Cross-sectional	Participants aged 20 years or older with complete anthropometric data, selected from IMSS medical units.	Participants who did not complete albuminuria measurement, had urinary tract infections, or did not attend the second measurement within three months.	3,901	50.6	49	Abdominal circumference was measured using a non-stretchable tape at the smallest circumference between the chest and hips.	≥90 cm for men; ≥80 cm for women.	Collected for the study.

Damaso (2024) (70)	Brazil	Cross-sectional	Women born between June 1978 and May 1979 in Ribeirão Preto, Brazil.	Pregnant women at the time of the study, and those without anthropometric data or incomplete obstetric questionnaire responses.	916	100	38	Abdominal circumference was measured at the midpoint between the last rib and the iliac crest with a measuring tape during exhalation.	≥88 cm for women.	Ribeirão Preto Birth Cohort 1978/79.
Gomes (2024) (71)	Brazil	Cross-sectional	Adults aged 20 years or older in rural areas of Pernambuco.	Pregnant women.	260	68.5	40.9	Measured with a measuring tape following WHO protocol.	≥90 cm for men; ≥80 cm for women.	IV State Survey on Health and Nutrition, Brazil.
