# Supplementary material for: Abdominal obesity prevalence in Latin America: a systematic review and meta-analysis comparing ATP III and IDF criteria
Source: Front Endocrinol (Lausanne). 2025 Jun 17;16:1562060. doi: 10.3389/fendo.2025.1562060 (PMC12208830; doi:10.3389/fendo.2025.1562060)
Supplement: Supplementary file 3 [file DataSheet3.pdf]

Supplementary Material 3. Risk of bias analysis of the selected studies

Author and Year	Was the sampling frame appropriate to address the target population?	Were the study participants recruited appropriately?	Was the sample size adequate?	Were the subjects and the study setting described in detail?	Was data analysis conducted with sufficient coverage of the identified sample?	Were valid methods used to identify the condition?	Was the condition measured in a standard and reliable manner for all participants?	Was the statistical analysis appropriate?	Was the response rate adequate, and if not, was the low response rate managed appropriately?	Total Score	Risk
Florez (2005)	*	*	*	*	*	*	*	*		8	Low
Marcopito (2005)	*	*	*	*	*		*	*		7	Low
Anselmo (2006)	*	*	*	*	*	*	*	*		8	Low
Mohanna (2006)	*	*		*	*	*	*	*		7	Low
Medina (2007)	*	*	*	*	*	*	*	*		8	Low
Perozzo (2008)	*	*	*	*	*	*	*	*		8	Low
Sá (2009)	*	*	*	*	*	*	*	*		8	Low
Cárdenas (2009)	*	*	*	*	*	*	*	*		8	Low
Parra (2009)	*	*	*	*	*	*	*	*		8	Low
Rojas (2010)	*	*	*	*	*		*	*		7	Low
Ferreira (2010)	*	*	*	*	*	*	*	*		8	Low
Patiño (2011)	*	*		*	*		*	*		6	Moderate
Marcal (2011)	*	*		*	*	*	*	*		7	Low

Guilherme (2011)	*	*	*	*	*	*	*	*		8	Low
Zarbato (2011)	*	*	*	*	*	*	*	*		8	Low
Bermúdez (2012)	*	*	*	*	*	*	*	*		8	Low
Da Silva (2012)	*	*	*	*	*	*	*	*		8	Low
Pérez (2012)	*	*	*	*	*	*	*	*		8	Low
Loret (2012)	*	*	*	*	*	*	*	*		8	Low
Sabino (2013)	*	*	*	*	*	*	*	*		8	Low
Bresciani (2013)	*	*	*	*	*	*	*	*		8	Low
Jiménez (2013)	*	*		*	*	*	*	*		7	Low
Rodrigues (2013)	*	*	*	*	*		*	*		7	Low
Salas (2014)	*	*	*	*	*	*	*	*		8	Low
Ibañez (2014)	*	*	*	*	*	*	*	*		8	Low
Costa (2014)	*	*		*	*	*	*	*		7	Low
Muñoz (2014)	*	*		*	*		*	*		6	Moderate
Alvarez (2014)	*	*	*	*	*	*	*	*		8	Low
Boing (2015)	*	*	*	*	*	*	*	*		8	Low
Orellana (2015)	*	*	*	*	*	*	*	*		8	Low
Dos Santos Costa (2016)	*	*	*	*	*	*	*	*		8	Low

Kumar (2016)	*	*	*	*	*		*	*		7	Low
Barengo (2016)	*	*	*	*	*		*	*		7	Low
Rosada (2016)	*	*	*	*	*		*	*		7	Low
Pires (2017)	*	*	*	*	*		*	*		7	Low
Orces (2017)	*	*	*	*	*	*	*	*		8	Low
Bernabé (2017)	*	*	*	*	*		*	*		7	Low
Mulatinho (2018)	*	*		*	*		*	*		6	Moderate
Pudla (2018)	*	*	*	*	*	*	*	*		8	Low
Vitorino (2018)	*	*	*	*	*		*	*		7	Low
Carrillo-Larco (2018)	*	*	*	*	*		*	*		7	Low
Martins-Silva (2019)	*	*	*	*	*		*	*		7	Low
Petermann (2019)	*	*	*	*	*		*	*		7	Low
Pajuelo (2019)	*	*	*	*	*		*	*		7	Low
Scherlowski (2020)	*	*	*	*	*	*	*	*		8	Low
Higuita (2020)	*	*	*	*	*		*	*		7	Low
Barranco (2020)	*	*	*	*	*		*	*		7	Low
Santana (2020)	*	*	*	*	*	*	*	*		8	Low
Ortiz (2021)	*	*	*	*	*	*	*	*		8	Low

De Sousa (2021)	*	*	*	*	*	*	*	*		8	Low
Bello (2021)	*	*	*	*	*		*	*		7	Low
Farro (2021)	*	*	*	*	*		*	*		7	Low
Aparco (2022)	*	*	*	*	*		*	*		7	Low
Campos-Nonato (2022)	*	*	*	*	*		*	*		7	Low
Rodrigues (2023)	*	*		*	*		*	*		6	Moderate
Do Nascimento (2023)	*	*		*	*		*	*		6	Moderate
Tavares (2023)	*	*	*	*	*		*	*		7	Low
Natal (2023)	*	*	*	*	*	*	*	*		8	Low
Alvarez (2024)	*	*	*	*	*	*	*	*		8	Low
Damaso (2024)	*	*	*	*	*	*	*	*		8	Low
Gomes (2024)	*	*		*	*	*	*	*		7	Low
