# Supplementary material for: Abdominal obesity prevalence in Latin America: a systematic review and meta-analysis comparing ATP III and IDF criteria
Source: Front Endocrinol (Lausanne). 2025 Jun 17;16:1562060. doi: 10.3389/fendo.2025.1562060 (PMC12208830; doi:10.3389/fendo.2025.1562060)
Supplement: Supplementary file 4 [file DataSheet4.pdf]

(A)

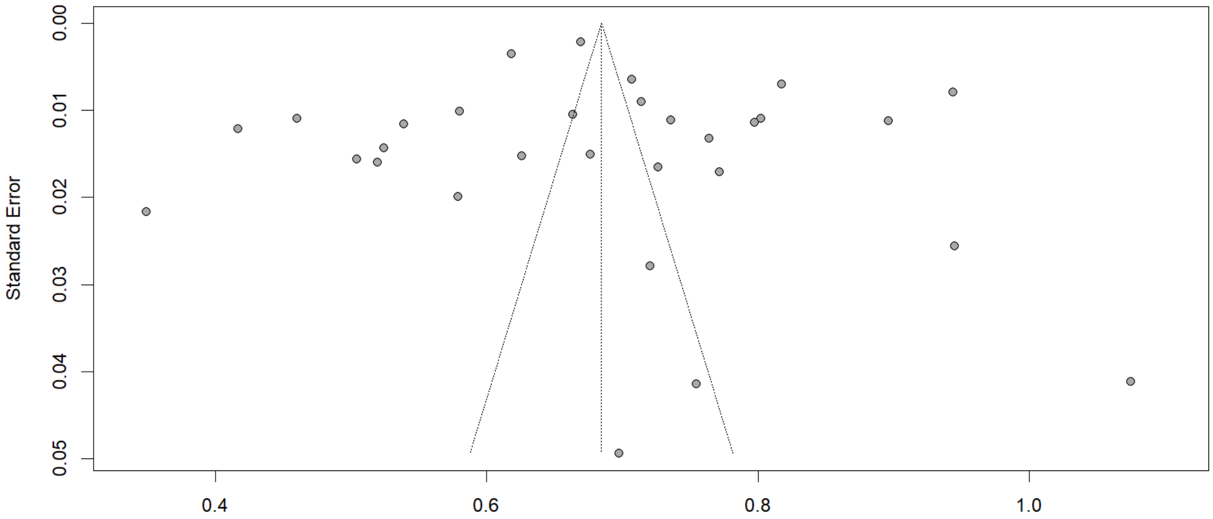

(B)

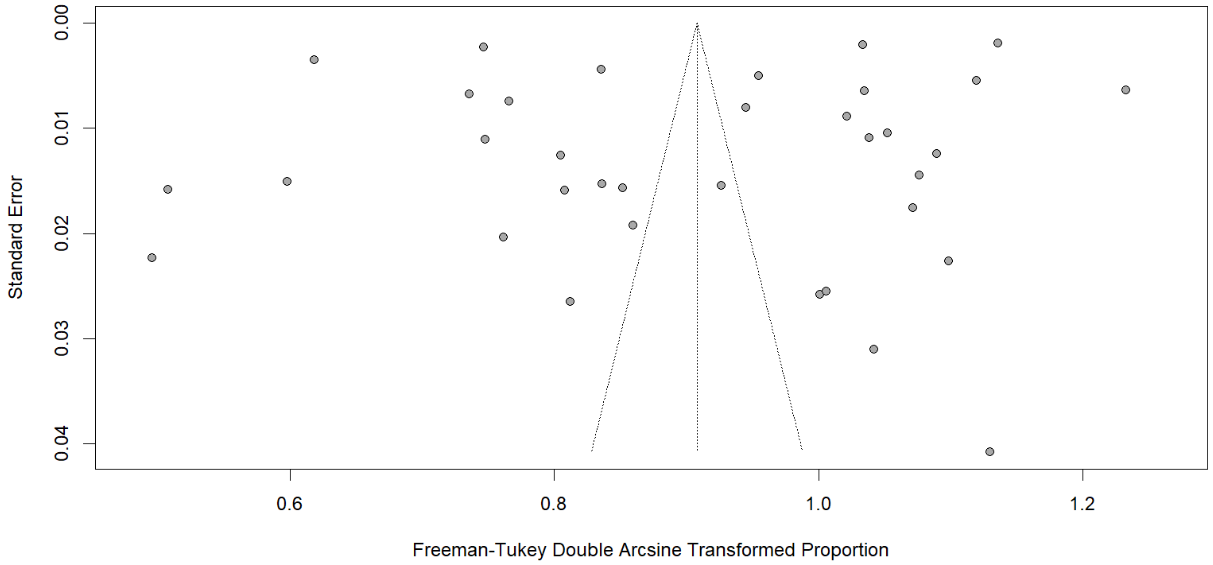

Supplementary material 4. Funnel plots for the assessment of publication bias in OA prevalence studies according to (A) ATP III and (B) IDF criteria in Latin America
