# Supplementary material for: Abdominal obesity prevalence in Latin America: a systematic review and meta-analysis comparing ATP III and IDF criteria
Source: Front Endocrinol (Lausanne). 2025 Jun 17;16:1562060. doi: 10.3389/fendo.2025.1562060 (PMC12208830; doi:10.3389/fendo.2025.1562060)
Supplement: Supplementary file 6 [file DataSheet6.pdf]

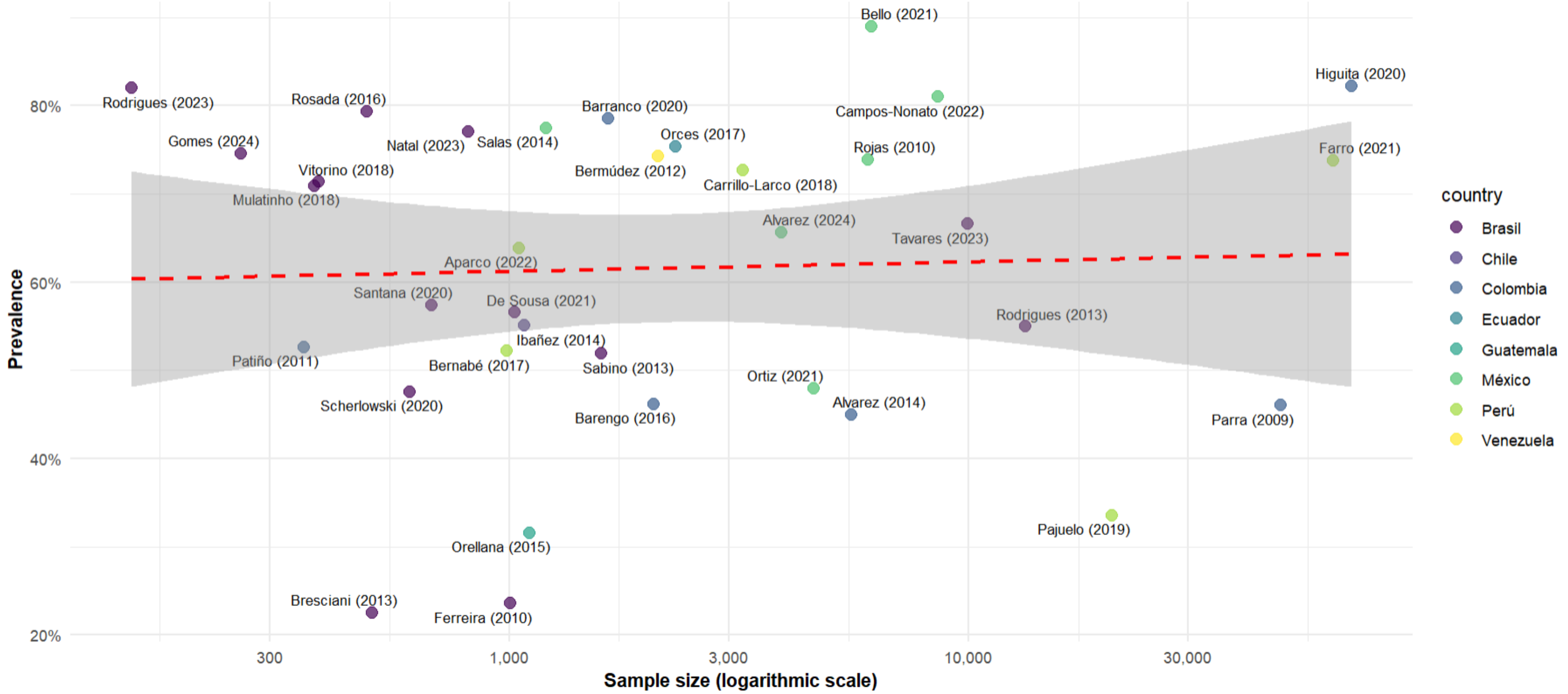

Supplementary material 6. Meta-regression of the prevalence of AO – IDF by year of sample size in Latin American countries
